# Genome-wide analysis of the endoplasmic reticulum stress response during lignocellulase production in *Neurospora crassa*

**DOI:** 10.1186/s13068-015-0248-5

**Published:** 2015-04-14

**Authors:** Feiyu Fan, Guoli Ma, Jingen Li, Qian Liu, Johan Philipp Benz, Chaoguang Tian, Yanhe Ma

**Affiliations:** Key Laboratory of Systems Microbial Biotechnology, Tianjin Institute of Industrial Biotechnology, Chinese Academy of Sciences, Xiqi Dao32, Tianjin Airport Economic Area, Tianjin 300308 China; University of Chinese Academy of Sciences, Yuquan Road, Beijing, 100049 China; Holzforschung München, TUM School of Life Sciences Weihenstephan, Technische Universität München, Hans-Carl-von-Carlowitz-Platz 2, Freising, Germany

**Keywords:** ER stress, UPR, Transcription factor, Lignocellulase secretion, RNA-seq, RESS

## Abstract

**Background:**

Lignocellulolytic fungal cells suffer endoplasmic reticulum (ER) stress during lignocellulase synthesis; however, an understanding of this integrated process on a genome-wide scale remains poor. Here, we undertook a systematic investigation of this process in *Neurospora crassa* (*N. crassa*) using transcriptomic analysis coupled with genetic screens*.*

**Results:**

A set of 766 genes was identified as the ER stress response targets (ESRTs) in *N. crassa* under cellulose utilization conditions. Among these, the expression of 223 and 186 genes showed dependence on IRE-1 and HAC-1, respectively. A total of 527 available mutants for ESRT genes were screened, 249 of which exhibited ER stress susceptibility, including 100 genes with unknown function. Disruption of *ire-1* or *hac-1* in *N. crassa* did not affect transcriptional induction of lignocellulase genes by cellulose but severely affected secretion of the corresponding enzymes. A global investigation of transcription factors (TFs) discovered three novel regulators (RES-1, RES-2, RRG-2) involved in lignocellulase secretion. Production of lignocellulases in *Δres-1* increased by more than 30% in comparison to wild type (WT), while secretion decreased by nearly 30% in strains *Δres-2* and *Δrrg-2*. Transcriptional profiling of the three TF mutants suggests they are deeply involved in lignocellulase secretion and ER stress response.

**Conclusions:**

Here, we determined the transcriptional scope of the ER stress response during lignocellulase synthesis in the model cellulolytic fungus *N. crassa.* Through genome-wide mutant screening and analysis, dozens of novel genes were discovered to be involved in the process. The findings of this work will be useful for strain improvement to facilitate lignocellulase and biomass-based chemical production.

**Electronic supplementary material:**

The online version of this article (doi:10.1186/s13068-015-0248-5) contains supplementary material, which is available to authorized users.

## Background

Saprophytic fungi evolved a highly efficient capability to secrete enzymes into their extracellular matrix to synergistically depolymerize biomass. This natural property has been exploited for lignocellulase production in industry [1]. Although *Trichoderma reesei* (*Hypocrea jecorina*) and *Aspergillus spp.* have been successfully developed as systems for lignocellulase production, a clear genetic basis underpinning the requirements of effective secretion has not yet been elucidated [2]. Early electron microscopy analysis revealed that endoplasmic reticulum (ER) proliferation occurred in the *T. reesei* hyper-secretion mutant RUT-C30 compared with its parental strain, QM6a [3,4]. Further characterization of the RUT-C30 strain revealed the transcript levels of ER-resident chaperone genes, such as *pdi1* and *bip1*, were induced in response to increased production of cellulase proteins [5]. These data suggested that the intracellular secretory pathway tightly regulates lignocellulase secretion in particular components of the ER.

The ER acts as the central hub where membrane and secretory proteins are properly folded and matured. When the ER encounters high protein flux or intractable heterologous proteins, its folding capacity could be transiently saturated, thus leading to a secretory pathway traffic jam and causing ER stress [6,7]. Cells activate multiple pathways to respond to ER stress, one of the best characterized being the unfolded protein response (UPR). In *Saccharomyces cerevisiae* and filamentous fungi, the UPR mainly depends on an evolutionarily conserved signaling cascade which is mediated by ER-resident transmembrane kinase/endoribonuclease IRE1 (Inositol-requiring enzyme-1) and the basic-leucine zipper (bZIP) transcription factor HAC1 (homologous to ATF/CREB1) [8]. Although several aspects of ER stress response and the UPR have been intensively unraveled using the *S. cerevisiae* model, filamentous fungi are capable of much higher protein production levels, suggesting different or additional response mechanisms might exist between the two systems. For instance, filamentous fungi utilize a feedback mechanism termed repression under secretion stress (RESS) [9-11] which selectively down-regulates transcription of genes encoding extracellular enzymes upon ER stress and thus helps to reduce ER load. However, the detailed mechanism behind RESS is not clear at the moment.

The Ascomycete *Neurospora crassa* is a common inhabitant of burnt plant material in nature and has recently become a model system to address lignocellulose deconstruction and utilization [12,13]. Previous transcriptome profiling of *N. crassa* grown on *Miscanthus* and cellulose revealed that the expression of most lignocellulase genes is significantly induced at early time points (16 h) but rapidly declines thereafter [14], implying RESS exists in *N. crassa* and might be a limiting step of lignocellulase synthesis. Moreover, recent work reported *hac-1* activation by unconventional mRNA splicing when *N. crassa* was grown on cellulose [15], suggesting the UPR cascade is needed to coordinate the increased lignocellulase flux and the cellular folding capacity. *N. crassa* has the unique advantage of a nearly complete genome deletion strain collection [16], which makes it an ideal resource for a systematic study of the ER stress response and lignocellulase synthesis. To our knowledge, studies on the cross-talk of these two processes have not been performed so far.

In this work, by using high-throughput mRNA sequencing technology (RNA-Seq) coupled to genetic screens, we explored the connection between fungal lignocellulase secretion and ER stress on a genome-wide level. We thus defined the regulon of the ER stress response and identified dozens of genes as well as three novel transcription factors: RES-1 (NCU03699), RES-2 (NCU02724), and RRG-2 (NCU02413) to be involved in this integrated process.

## Results

### The experimental design setup for the analysis of mild and acute ER stress during cellulase production in *N. crassa*

**Figure 1:**
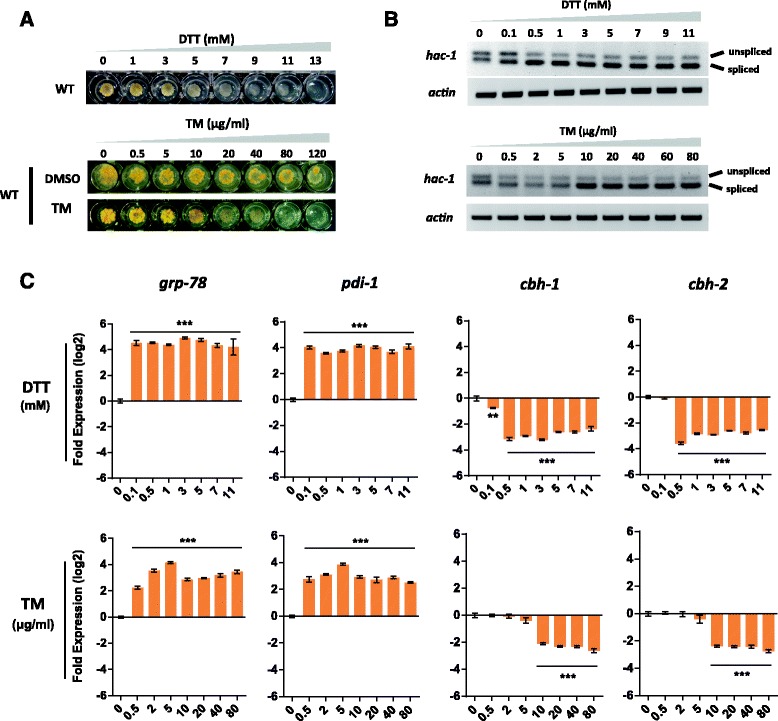
Defining applicable ER stress stimulation conditions. ER stress was induced by incubating *N. crassa* in the presence of concentration gradients of dithiothreitol (DTT) or tunicamycin (TM), and several criteria were used to define ER stress levels, including (**A**) sensitivity of growth to the drugs. WT conidia were inoculated onto solid GFS agar in a multi-well plate containing the indicated concentrations of DTT or TM and incubated at 25°C for 3 days. (**B**) Monitoring *hac-1* non-canonical splicing using semi-quantitative RT-PCR. WT conidia were inoculated into Vogel’s medium with Avicel as carbon source for 36 h, then either DTT or TM was added at different concentrations as indicated for an extra 1 h before harvesting. The product sizes representing *hac-1* spliced and un-spliced forms were 201 bp and 224 bp, respectively. The gene *actin* (NCU04173) was used as a loading control. (**C**) Monitoring UPR and RESS activation by qPCR. Culture conditions were as in (**B**). The data are normalized to expression on mock = 1 and re-calculated to log_2_-fold (standard error of the mean (SEM), *n* = 3). The asterisk indicates a significant difference from mock (**P < 0.01, ***P < 0.001) via one-way ANOVA.

To trigger ER stress while eliminating any non-specific drug response noise, the *N. crassa* wild-type (WT; FGSC#2489, *Mat A)* was treated with two kinds of ER stress-causing agents: either dithiothreitol (DTT; prevents disulfide bond formation,) or tunicamycin (TM; inhibits *N*-linked glycosylation). Cells can dynamically activate distinct physiological processes, such as adaptation, alarm, or apoptosis, depending on the ER stress intensity [7,17]. In order to maximize the detection of targets which contribute to ER stress resistance and minimize apoptotic effects, three experiments were initially employed to determine the appropriate treatment (Figure 1). When the sensitivity of *N. crassa* WT to DTT and TM was measured using concentration gradients (Figure 1A), we observed a range of growth phenotypes from mild inhibition at low concentrations (1 to 3 mM DTT or 0.5 to 10 μg/mL TM), to significant impairment at moderate concentrations (5 to 11 mM DTT or 20 to 80 μg/mL TM) and complete inability to grow when the conidia were exposed to 13 mM DTT or 120 μg/mL TM, respectively. We considered these results to reflect lethal, acute, and mild stress levels. We next checked the IRE-1-mediated mRNA splicing of the unfolded protein response marker *hac-1* [15] by treating *N. crassa* cells with a similar concentration gradient of either DTT or TM during growth on cellulose. The results shown in Figure 1B suggest that a treatment with 0.1 mM DTT or 5 μg/mL TM is sufficient to achieve mild stress levels. Finally, to determine the impact of distinct stress levels on lignocellulase synthesis, we measured the expression levels of two well-known UPR maker genes, *grp-78* (NCU03928) and *pdi-1* (NCU09223), as well as two major cellobiohydrolase genes, *cbh-1* (NCU07340) and *cbh-2*(NCU09680), by quantitative PCR (qPCR) (Figure 1C). We found the folding machinery to be activated at all tested stress levels, while *cbh-1* and *cbh-2* were dramatically down-regulated at concentrations exceeding 0.1 mM DTT or 5 μg/mL TM, respectively, suggesting an induction of RESS to render cells more resistant against acute ER stress. Thus, based on growth phenotype, the splicing pattern of *hac-1* mRNA and RESS mediated down-regulation of cellulase genes, four stress conditions were finally chosen for further analysis: mild ER stress by treatment with 0.1 mM DTT or 5 μg/mL TM; and acute ER stress by treatment with 5 mM DTT or 40 μg/mL TM.

### Defining the ER stress response targets in wild-type *N. crassa* during cellulase production

**Figure 2:**
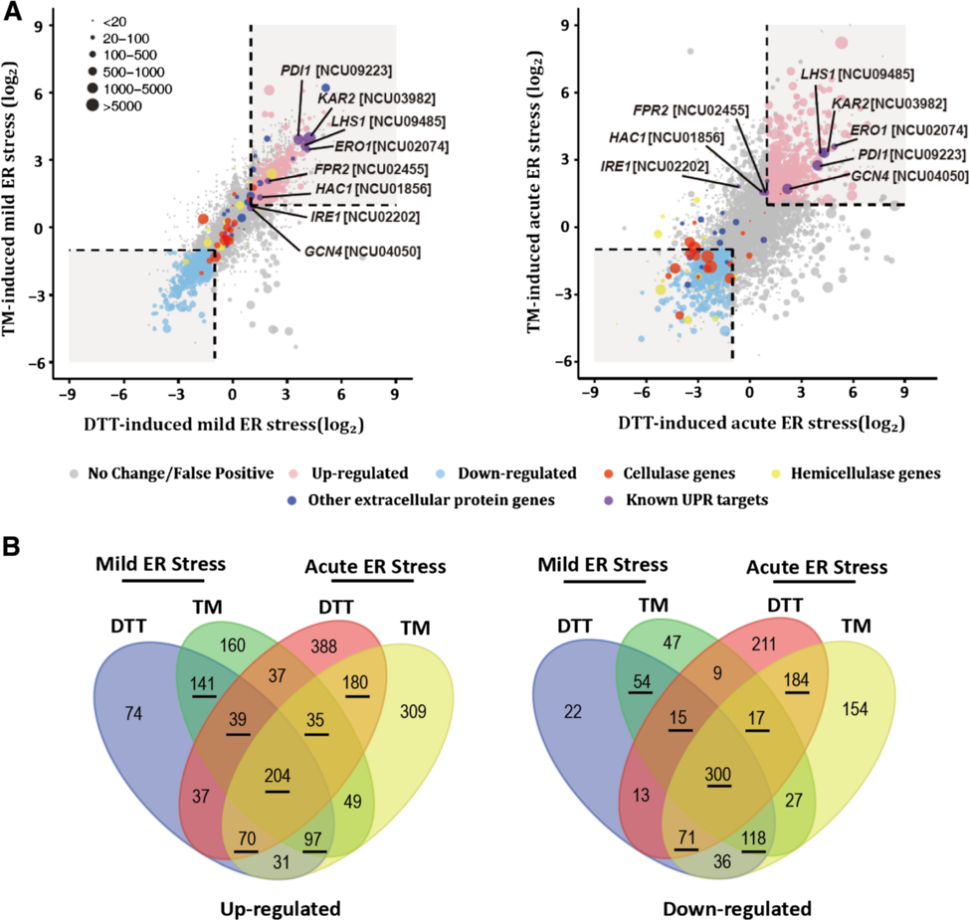
Panorama of ER stress response in *N. crassa.* (**A**) The pattern of intensity-dependent ER stress response (left: mild stress induced by 0.1 mM DTT or 5 μg/mL TM; right: acute stress induced by 5 mM DTT or 40 μg/mL TM) was plotted by calculating the log_2_ normalized fold changes in wild-type cells treated with DTT or TM *versus* the mock treatment. Each point represents a transcript with a detected expression level. Points filled with light-pink or light-blue represent genes expression with increased or decreased abundance (*q* value ≥0.95) upon ER stress. Gray points represent genes with no differences in the expression or false positives (see the ‘Materials and methods’ section for detail). Cellulases (red points), hemicellulases (yellow points), and extracellular proteins (dark blue points) previously identified by LC-MS [14] are indicated to show the RESS. The black dotted line represents a manually defined threshold (|log_2_ ratio| = 1). The mean of *D* value (the difference of RPKM for each gene between two samples in each comparison) of two comparisons (DTT and TM) from NOISeq results were plotted and the size of dots reflects a given level of transcript abundance. The locations of eight known UPR targets in the diagram are indicated to demonstrate that UPR was elicited successfully. (**B**) Four-way Venn diagram pooling genes according to their expression response to the four different treatments. Marked (black bar) are the 766 ESRTs up-regulated by both DTT and TM at either mild or acute ER stress levels (left) and the 759 down-regulated targets upon ER stress (right).

Comparative analyses of the transcriptional profiling data of WT under four ER stress conditions (mild ER stress and acute ER stress by treatment with DTT or TM as described above) *versus* the no drug condition were performed. Of the 9,730 annotated genes in the *N. crassa* genome, we detected 1,322 (13.59%), 1,349 (13.86%), 1,810 (18.60%), and 1,882 (19.34%) genes with altered expression which determined by NOISeq (for detail of cut-off, see the ‘Materials and methods’ section) for each of the four conditions, respectively (Additional file 1: Table S1 p1 and Additional file 2: Table S2 p1-4). The global regulation patterns during mild and acute ER stresses were graphed separately (Figure 2A). Elevated expression levels in a subset of 766 (7.87%) genes induced by both DTT and TM at either mild or acute ER stress levels were deemed to be the result of true ER stress rather than non-specific effects and therefore defined as the ER stress response targets (ESRTs: 277 mild-specific targets, 285 acute-specific targets, and 204 genes generally up-regulated under both types of stress) (Figure 2B, Additional file 3: Table S3 p1, p3). Several well-established UPR targets [18] were found to be included in our ESRT dataset (Figure 2A). This result was confirmed by qPCR assays using independent biological samples, showing consistency with the RNA-seq data (Additional file 4: Figure S1). Furthermore, we also collected 759 (7.80%) genes (Figure 2B) with decreased abundance upon ER stress (Figure 2B and Additional file 3: Table S3 p4, p6). Including cellobiohydrolase-1 (*cbh-1,* NCU07340) and cellobiohydrolase-2 (*cbh-2,* NCU09680), a total of 23 major lignocellulase genes were found to belong to this dataset and be specifically down-regulated under acute ER stress (Figure 2A and Additional file 3: Table S3 p7), implying that a rapid correction of intracellular transcript pools is needed to tolerate acute stress.

Further functional categorization of 462 (60.31%) annotated genes of the 766 ESRTs using FunCat [19] in addition to manual classification based on Pfam domain annotations and BLAST hits showed most of them were linked to protein secretion or the biogenesis of secretory organelles (Additional file 3: Table S3 p2, p3). Overrepresented categories found were for example as follows: protein folding, modification, destination (155 genes, *P* = 1.49e-07), membrane lipid metabolism (19 genes, *P* = 3.89e-03), transport facilities and transport routes (166 genes, *P* = 1.86e-09), as well as cell growth/morphogenesis (41 genes, *P* = 4.52e-03). Extending previous observations, the expression pattern of certain functional gene groups were found to depend on stress intensity. For example, genes encoding members of the p24 family (for example, NCU01342 [*ERV25* in *S. cerevisiae*], NCU03800 [*ERP3*], and NCU04003 [*ERP1*]), which are involved in ER to Golgi transport, were specifically up-regulated upon mild stress levels. In contrast, genes encoding most components of COPII transport vesicles involved in ER to Golgi transport were generally up-regulated under any stress levels. In addition, the expression levels of half of the genes engaged in *N*-linked or *O*-linked glycosylation (13 of 26) were enhanced solely under mild stress conditions whereas most genes related to phospholipid metabolism (9 of 13) were only up-regulated upon acute stress intensity (Additional file 3: Table S3 p3). Furthermore, our ESRT dataset included several complete complexes within the secretory pathway, such as the SEC61 translocon complex (NCU08897 [*SEC61* in *S. cerevisiae*], NCU08379 [*SBH1/SBH2*], and NCU04127 [*SSS1*]), the SEC63 complex (NCU00169 [*SEC63*], NCU06333 [*SEC62*], NCU02681 [*SEC66*], and NCU07746 [*SEC72*]), as well as the SNARE complex involved in vesicle-mediated Golgi to ER retrograde trafficking (NCU06708 [*SEC22*], NCU00953 [*SEC20*], NCU07939 [*UFE1*], and NCU00184 [*USE1*]). Given that around 40% of genes in the *N. crassa* genome are without clear function, the ESRTs identified here will aid in detecting novel component function in secretory pathway of filamentous fungi.

### Genome-wide overview of the ESRT deletion mutants in *N. crassa*

**Figure 3:**
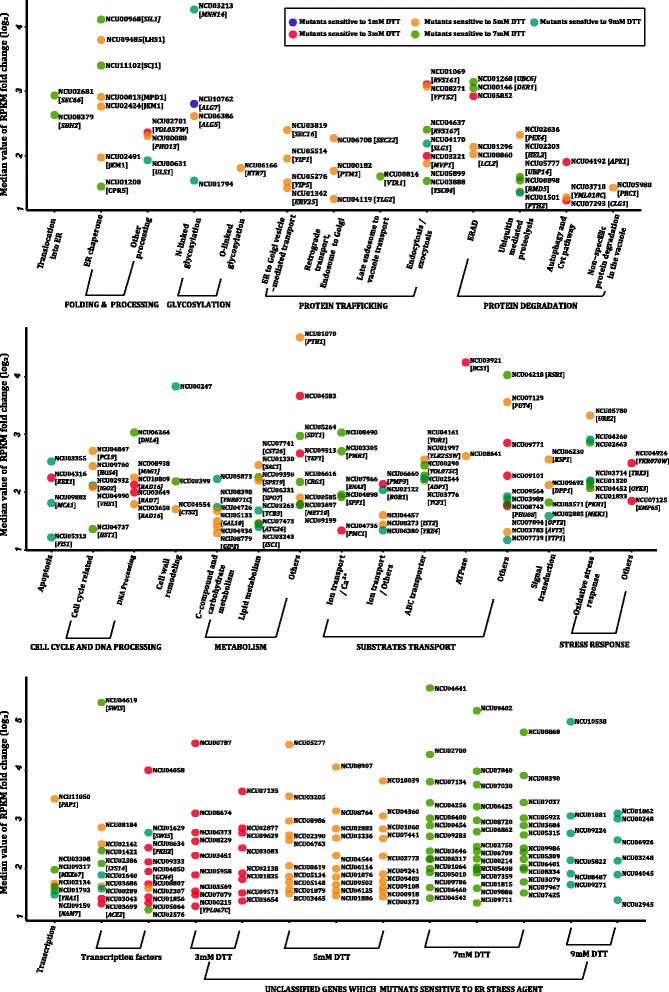
Expression induction and functional categorization of 249 *N. crassa* KO mutants which exhibited ER stress susceptibility. Each point on the scatter plot represents the median value of fold changes of WT on mild DTT, mild TM, acute DTT, and acute TM (log_2_ normalized, the full data see Additional file 3: Table S3, p3) and functional category of an ESRT gene whose KO mutants exhibited ER stress susceptibility. DTT-sensitive phenotypes are plotted using different colors, from strongest to weakest along a gradient of dark-purple (sensitive to 1 mM DTT), red (3 mM), orange (5 mM), green (7 mM) to cyan (9 mM).

To provide a basic understanding of the function of ESRT genes discovered by transcriptome analysis, we phenotypically screened 527 mutants of the total 766 ESRT targets (including 526 homokaryotic gene knock-out strains and one single-base-pair deletion mutant for *cpc-1* which results in a nonfunctional, truncated polypeptide of this amino acid regulator [20]) and 249 genes that appear to be pivotal for ER stress resistance in the WT (Figure 3) for their sensitivity to ER stress (Additional file 5: Table S4). Most mutants were able to survive in the presence of 1 mM DTT, except for strain *ΔNCU10762*. In *S. cerevisia*e, the homolog of NCU10762 is *ALG7*, which encodes the UDP-N-acetyl-glucosamine-1-P transferase (GPT) that transfers the first N-acetylglucosamine to dolichyl-phosphate in the first step of the dolichol pathway of *N*-linked protein glycosylation [21]. Moreover, we found another 248 genes whose deletion caused the *N. crassa* cells to become DTT-sensitive, with 44, 84, 82, and 38 of them sensitive to 3, 5, 7, and 9 mM DTT, respectively. As anticipated, many of these genes had roles in the secretory pathway, such as genes involved in protein translocation into the ER (for example, NCU02681 [*SEC66* in *S. cerevisiae*], NCU08379 [*SEC61*], protein folding (for example, NCU00813 [*MPD1*], NCU09485 [*LHS1*], NCU11102 [*SCJ1*], NCU00968 [*SIL1*]), ER-associated degradation (ERAD) (for example, NCU00146 [*DER1*], NCU01296 [*DER1*], NCU01268 [*UBC6*]), protein glycosylation (for example, NCU06386 [*ALG5*], NCU06166 [*KTR7*]), and protein trafficking (for example, NCU03819 [*SEC16*], NCU01342 [*ERV25*], NCU05514 [*YIP1*], NCU06708 [*SEC22*]). However, many mutants for genes with pivotal functions as unfolded protein response targets could not be assayed because they lacked homokaryotic mutants, such as *grp-78* (NCU03982) and *pdi-1* (NCU09223), as well as nearly half of the genes engaged in protein translocation, glycosylation, and trafficking (Additional file 3: Table S3 p3). It is likely that these genes are essential for cell viability.

Importantly, our screen provided functional data that 100 (13.05%) formerly unclassified genes could be directly or indirectly involved in ER stress. To test whether these ESRTs also contribute to cellulase secretion, we next checked the cellulase production of 18 mutants sensitive to 3 mM DTT and 31 mutants with sensitivity to 5 mM DTT in liquid batch culture. Deletion of five genes, namely NCU07135, NCU08986, NCU04360, NCU01886, and NCU00918, decreased cellulase secretion titers by more than 30% when compared with the WT (Additional file 4: Figure S2), suggesting the products of those five genes might function in the secretory pathway. The function of the other candidates remains unknown, although the DTT-sensitive phenotype suggests they might be involved in the ER stress response.

### Identification of ER stress response targets that are regulated by the canonical IRE-1/HAC-1 UPR pathway

**Figure 4:**
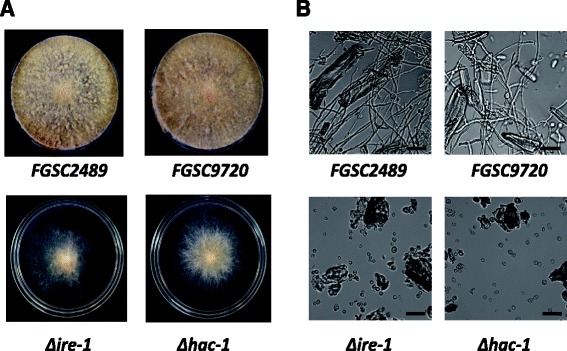
Deletion of *ire-1* and *hac-1* affects cell growth and causes cellulose utilization defects. (**A**) Comparison of radial growth phenotypes of *ire-1* and *hac-1* with their parental strain. Conidia (around 10^3^) were dotted on minimal medium (MM) plates, grown for 72 h at 25°C. (**B**) The conidia of *Δire-1* and *Δhac-1* mutants fail to germinate under cellulolytic induction conditions (presence of Avicel as sole C-source). Pictures were taken after 36 h of culture; the irregular blocks in the images represent Avicel particles. The black bar indicates a length of 20 μm.

As in *S. cerevisiae*, no clear homologs to ER stress sensors such as PKR-like ER kinase (PERK) and activating transcription factor-6 (ATF6) were found in *N. crassa*, implying the IRE-1 (encoded by NCU02202) and HAC-1 (NCU01856) mediated pathway might be the only route to trigger the UPR cascade. In comparison to the homologs from other species [22-24], the *N. crassa hac-1* mRNA contains an atypical intron (23 nt) [15] similar in size to higher eukaryotes, such as *Arabidopsis thaliana* (23 nt), *Drosophila melanogaster* (23 nt), and *Homo sapiens* (26 nt), rather than *S. cerevisiae* (252 nt) (Additional file 4: Figure S3A), revealing an intimate phylogenetic link between higher eukaryotes and filamentous fungi. To explore how the IRE-1/HAC-1 mediated UPR pathway affects ER stress response and lignocellulase production, a KO mutant for *hac-1* was constructed while *Δire-1* was purified by sexual crossing (Additional file 4: Figure S3B,C,D). Consistent with previous observations in *Aspergillus fumigatus* [25] and other eukaryotes [22,23], a loss of IRE-1 was sufficient to impair the unconventional splicing of *hac-1* mRNA in *N. crassa* (Additional file 4: Figure S3E). In comparison to their parental strains, both *Δire-1* and *Δhac-1* mutants showed a marked reduction (approximately 32%) in radial growth on solid minimal medium (Figure 4A). Deletion of either *ire-1* or *hac-1* did not visibly affect the width (approximately 5 μm) of the middle intercalary cell of conidia in *N. crassa* (Additional file 4: Figure S4A); however, during germination, huge vacuoles accumulated within young hyphae of both mutants, probably caused by a vesicle trafficking malfunction (Additional file 4: Figure S4B). In liquid batch culture using microcrystalline cellulose (Avicel) as the sole carbon source, more than 97% of both *Δire-1* and *Δhac-1* conidia failed to germinate (Figure 4B), suggesting UPR is a key factor for the cellulase secretion capacity of *N. crassa*.

We further explored which ESRTs were regulated by the UPR pathway through transcriptome profiling of both *Δire-1* and *Δhac-1* strains (Additional file 1: Table S1 p2 and Additional file 2: Table S2 p5-10). Because these strains are unable to use cellulose, sucrose pre-grown mycelia were used for the profiling analysis instead of directly inoculating cultures with conidia. Bioinformatic analysis (Additional file 6: Supporting information) revealed that 223 and 186 genes out of the 766 ESRTs were regulated by IRE-1 and HAC-1, respectively (Additional file 7: Table S5 p5). As expected from previous observations in *S. cerevisiae* [18,26] and other model systems [11,25,27-29], the IRE-1/HAC-1 governed UPR regulon mainly functions to facilitate protein folding and to remodel the secretory pathway. Major enriched functional categories (FunCat) were protein folding and stabilization (*P* = 4.44e-06 for IRE-1, *P* = 3.72e-06 for HAC-1), glycosylation (*P* = 1.15e-11 for IRE-1, *P* = 7.07e-13 for HAC-1) as well as vesicular transport (Golgi network, etc.) (*P* = 6.35e-24 for IRE-1, *P* = 5.11e-22 for HAC-1). Although *Δire-1* and *Δhac-*1 failed to grow on Avicel, lignocellulase genes were found to be induced to similar levels compared with their parental strains (Additional file 1: Table S1 p2), suggesting the UPR pathway does not directly regulate the induction of hydrolytic enzyme genes by cellulose. In addition, the expression levels for most lignocellulase genes were markedly down-regulated when acute ER stress emerged in both the *Δire-1* and *Δhac-1* backgrounds (Additional file 1: Table S1 p2 and Additional file 2: Table S2 p6, p8), implying RESS is independent of the UPR pathway. Furthermore, the analysis of intracellular cellulase synthesis and secretory pathway protein loading in *Δire-1* and *Δhac-*1 revealed that the cellulase synthesis in both mutants was similar to the parental strains (after a culture switch from sucrose to cellulose for 4 h and 36 h). Therefore, the observed reduction in secreted cellulases implies a reduced secretion capacity within this time frame. At a later stage (96 h), the amount of intracellular cellulases was diminished, which might be a result from a negative feedback-loop of cellulase hypo-secretion (Additional file 4: Figure S5A,B). These observations demonstrate that IRE-1 and HAC-1 play an important role in the *N. crassa* cellulase secretion system.

### Systematic characterization of TFs required for ER stress response during cellulase synthesis

**Figure 5:**
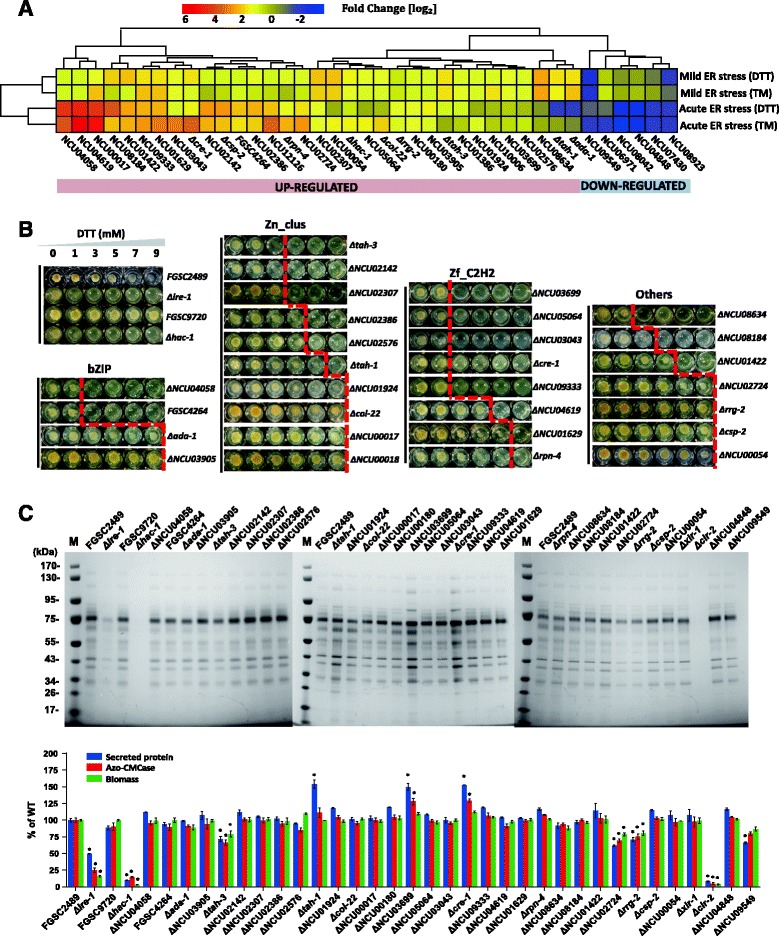
Screen for transcription factors involved in ER stress response and cellulase synthesis. (**A**) Heatmap of 39 transcription factors (TFs) found to be differentially expressed in response to ER stress under distinct stress intensities: 33 up-regulated and 6 down-regulated. The degree of expression abundance was normalized by RPKM-fold change (log_2_). Names of TF DNA-binding domains according to Pfam annotation were indicated in square brackets. (**B**) Screening of DTT susceptibility of 30 available ESRT TF KO mutants. The red line indicates the growth to no growth threshold and is an indicator for sensitivity to ER stress elicited by DTT. (**C**) Batch cultures of ESRT TF KO mutants were prepared to discover targets affecting cellulase secretion. After 7 days of culture with rich nitrogen source, the typical secretome of each TF candidate is shown on SDS-PAGE gel (upper panel), while total extracellular protein concentration, CMCase activity, and biomass (as total protein) were measured and displayed after normalization to the WT control (lower panel) by percentage (standard error of the mean (SEM), *n* = 3, the asterisk (*) indicates a significant difference from WT with an unadjusted *P* value of <0.001 using a one-way ANOVA).

For both ER stress response and cellulase synthesis, transcription factor (TF) mediated orchestration of gene expression is crucial. We systematically searched for TFs with significantly altered expression patterns in our recorded differential expression data based on acute/mild ER stress (Figure 2). Of the 266 predicted TFs present in the *N. crassa* genome (based on Broad version 7) [30] (Additional file 8: Table S6 p1-2), 33 were up-regulated in the presence of ER stress, while 6 TFs were down-regulated (Figure 5A and Additional file 8: Table S6 p3).

Of the 33 TFs with increased abundance upon ER stress, five (15.15%) belonged to the basic-region leucine zipper (bZIP) family, 11 (33.33%) to the fungal Zn(2)-Cys(6) binuclear cluster family, nine (27.27%) to the Cys2His2 (C2H2)-type zinc fingers, and eight to miscellaneous families (24.24%) (Additional file 8: Table S6 p3). We next screened 30 available homokaryotic deletion mutants out of 33 TFs for DTT-sensitivity; compared with WT, 20 TFs exhibited significant DTT-sensitive phenotypes, including the UPR regulator *hac-1* (NCU01856) (Figure 5B). Additionally, six TFs were found to be regulated in an IRE-1/HAC-1 mediated manner; including four TFs (NCU09333, NCU03043, NCU03686, NCU02386) affected by IRE-1 and two TFs (NCU01422, NCU06095) that were dependent on HAC-1 (Additional file 7: Table S5 p5).

To assess whether those TFs regulated by ER stress also contribute to cellulase secretion, liquid batch cultures with (Figure 5C) or without (Additional file 4: Figure S6) a rich nitrogen source were performed. The *Δire-1* and *Δhac-1* strains, along with their parental strains, were included as controls. Consistent with previous observations [31], the *Δcre-1* strain displayed an increase of approximately 50% in the amount of secreted protein compared to the WT strain. A loss of *ire-1* led to a 50% decrease of the secreted protein levels in comparison to the WT strain, while the *Δhac-1* KO mutant almost completely lost its ability to produce lignocellulases. Using ±30% of secreted proteins produced by WT as a threshold (refer to the *Δcre-1* published data [31]) and considering the data from both nitrogen conditions, we found *ΔNCU03699* could significantly promote lignocellulase secretion similar to *Δcre-1*, while *ΔNCU02413* (*Δrrg-2*) and *ΔNCU02724* exhibited repressive effects. The intracellular cellulase loading of the secretory pathway was further examined in these mutants using switch experiments of sucrose pre-grown strains shifted to Avicel. While the intracellular cellulase production was not affected until after 96 h (Additional file 4: Figure S5A,B), extracellular cellulase accumulation was substantially increased (*ΔNCU03699*) or reduced (*ΔNCU02413* (*Δrrg-2*) and *ΔNCU02724*) (Additional file 4: Figure S7A,B), implying that the secretion capacity rather than cellulase synthesis is affected in these strains. These findings suggest that the hyper- or hypo-secretion phenotype of these mutants is linked to cellulase secretion (Additional file 4: Figure S7A,B). To the best of our knowledge, this is the first report providing strong functional data indicating that these TFs are involved in lignocellulase production and ER stress, although NCU02413 (*rrg-2*) had previously been suggested to play a role in the oxidative stress response in *N. crassa* [32]. Based on their involvement in the overall process, we named the TFs encoded by NCU03699 and NCU02724 RES-1 and regulator for ER stress response (RES-2), respectively.

Of the six TFs with decreased abundance upon ER stress, three belong to the fungal Zn(2)-Cys(6) binuclear cluster domain family, including the two core regulators of plant cell wall degradation: CLR-2 [33] and XLR-1 [34]. Because most lignocellulase genes that are down-regulated in the presence of acute ER stress belong to the CLR-2 or XLR-1 regulons, it seems likely that RESS results to a great extent from the down-regulation of these core regulators. Two of the other four TFs that were found to be down-regulated under ER stress (NCU09549, NCU04848, NCU08923, and NCU07430 had available mutants that allowed us to test their enzyme production. Slightly decreased lignocellulase production was recorded for ΔNCU09549 when compared to WT, while no obvious defect was observed in the ΔNCU04848 mutant (Figure 5C).

### RES-1, RES-2, and RRG-2 are required for ER stress response and cellulase synthesis

**Figure 6:**
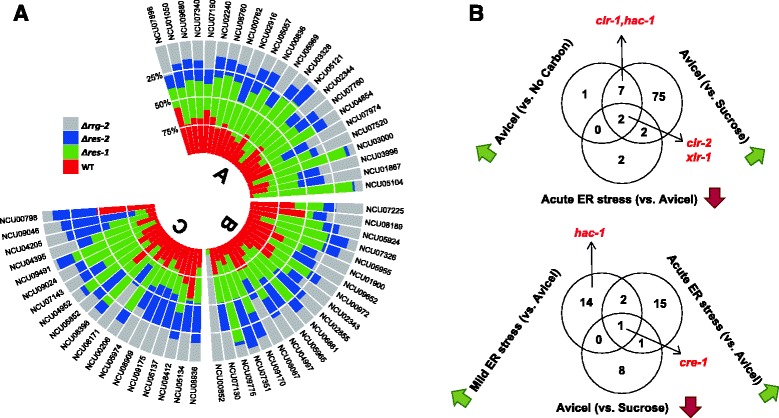
RES-1, RES-2, and RRG-2 indirectly affect lignocellulase gene expression. (**A**) Comparison of lignocellulase expression levels in *Δres-1*, *Δres-2*, and *Δrrg-2* with WT. A total of 62 genes were grouped into three clusters: [A] cellulase genes, [B] hemicellulase genes, and [C] other genes encoding extracellular proteins. For each target, the relative proportion of transcript abundance (RPKM) is indicated as a percentage of the total. (**B**) Three-way Venn diagrams generated to search for TFs, which are coordinately expressed with lignocellulase genes. Top diagram: TFs induced (green arrow) by Avicel *versus* no carbon (top left) and *versus* sucrose condition (top right) and repressed (red arrow) under acute ER stress (bottom) were reasoned to potentially have a positive effect on lignocellulase gene expression. Bottom diagram: In addition, TFs being repressed when exposed to Avicel *versus* sucrose treatment while being induced upon ER stress could likely exert a negative effect on lignocellulase gene expression.

Since RES-1, RES-2, and RRG-2 likely function to bridge the ER stress response to lignocellulase expression, transcriptional profiling of the three corresponding TF deletion strains was performed under ER stress conditions. Deletion of *res-1* (encoded by NCU03699) resulted in up-regulation of 583 genes in comparison to WT in cells grown on Avicel for 36 h. Consistent with the protein secretion data (Figure 5C), FunCat analysis of the 583 potential *res-1* targets showed 38 genes belonging to the ‘polysaccharide metabolism’ group, with many lignocellulase genes being enriched (*P* = 6.11e-09) (Figure 6A and Additional file 9: Table S7 p1). For example, the gene expression of several *AA9* family members such as NCU08760 (*AA9-5*), NCU03328 (*AA9-6*), NCU00836 (*AA9-7*), NCU03000 (*AA9-8*) as well as some other glycosyl hydrolases such as NCU05121 (*gh45-1*, endoglucanase V), NCU06861 (*gh43-3*), NCU07326 (*gh43-6*), and NCU04952 (*gh3-4*, β-glucosidase) showed a significant increase in the *Δres-1* strain compared to WT. The expression levels of NCU09664 and NCU04870, encoding acetylxylan esterases, increased more than 33- and 7-fold, respectively, and a gene encoding the extracellular feruloyl esterase B (NCU09491) increased over 30-fold upon loss of RES-1. Surprisingly, the expression of several major cellulase genes, such as NCU07340 (*cbh-1)*, NCU09680 (*cbh-2*), and NCU00762 (*gh5-1*, endoglucanase 3), was WT-like in *Δres-1* after growth on Avicel for 36 h, although significant hyper-secretion was observed after 7 days of culture (Figure 5C), suggesting RES-1 could affect lignocellulase production not only at the transcriptional but also at the post-transcriptional level as protein, thus the latter effect is exerted indirectly. In good agreement with this hypothesis, we found that a deletion of *res-1* resulted in 76 ESRTs failing to be up-regulated upon exposure to DTT (Additional file 9: Table S7 p2-3), especially genes functioning in glycosylation (*P* = 2.8e-03, for example, NCU02541 and NCU03995, which separately encode the oligosaccharyltransferase alpha and gamma subunit-like proteins) as well as lysosomal and vacuolar protein degradation (*P* = 5.38e-05, for example, NCU01955 [autophagy-related protein 3], NCU02515 [dipeptidyl aminopeptidase]).

The *Δres-2* and *Δrrg-2* deletion strains exhibited repressive effects on lignocellulase secretion. Transcriptome profiles showed deletion of *res-2* specifically down-regulated 491 genes compared with WT when cells were grown on Avicel, including 87 genes involved in C-compound and carbohydrate metabolism (*P* = 1.62e-06) (Figure 6A and Additional file 9: Table S7 p4). For instance, hemicellulase genes such as NCU07225 (*gh11-2,* endo-1, 4-beta-xylanase 2), NCU08189 (*gh10-2,* endo-1,4-beta-xylanase), and NCU00972 (*gh53-1*, arabinogalactan endo-1,4-β-galactosidase) decreased more than three- to fourfold under Avicel conditions in the *Δres-2* mutant as compared to WT. We noticed the major cellulase genes such as NCU07340 (*cbh-1*), NCU09680 (*cbh-2*), and NCU00762 (*gh5-1*) also showed a 1.6 ~ 1.8-fold decrease in expression under Avicel conditions in the *Δres-2* strain, although the respective data did not pass the *Q* value cut-off. In addition, 118 ESRTs were shown to be regulated by the TF RES-2, including targets involved in cellular export and secretion (*P* = 7.68e-03), cellular import (*P* = 5.49e-03), enzyme inhibitors (*P* = 7.10e-03), as well as several targets functioning in autophagy and cytoplasm to vacuole-targeting (Cvt) pathways, such as NCU01955 (autophagy-related protein 3), NCU04192 (vacuolar aspartyl aminopeptidase Lap4), and NCU01545 (autophagy protein 8) (Additional file 9: Table S7 p5-6).

Similar to *res-2*, a deletion of *rrg-2* down-regulated 102 genes compared with WT when cells were grown on Avicel, including 25 genes involved in C-compound and carbohydrate metabolism (*P* = 6.11e-05) (Figure 6A and Additional file 9: Table S7 p7), such as lignocellulase genes NCU09680 (*cbh2*), NCU02240 (*AA9-1*, endoglucanase II), and NCU05955 (xyloglucanase). The expression of genes encoding glucoamylases was also found to be down-regulated (for example NCU01517 and NCU08746). In addition, *rrg-2* also appeared to regulate 115 ESRTs related to lipid, fatty acid, and isoprenoid metabolism (*P* = 4.90e-03), glycosylation (*P* = 1.97e-03), ER to Golgi transport (*P* = 9.05e-03), etc. (Additional file 9: Table S7 p8-9).

Lignocellulase genes can be induced significantly when *Neurospora* is exposed to cellulose; however, even in the presence of a cellulosic inducer, these genes can also be rapidly down-regulated when suffering RESS. Therefore, TFs with a similar expression pattern to lignocellulase genes under both physiological conditions are likely to be involved in cellulase regulation. We re-analyzed published transcriptome profiling data of *N. crassa* grown on sucrose, Avicel, and no carbon (starvation) and compared these with our ER stress response data [35] (Additional file 8: Table S6 p3-4; Figure 6B). Ten and 86 TFs were significantly induced by Avicel compared to either the no carbon or sucrose controls, respectively (Figure 6B, upper panel); nine of them were up-regulated in both conditions. Besides *clr-1*, *clr-*2, and *xlr-1*, *hac-1* was also included, suggesting lignocellulase induction and UPR activation act synergistically. Filtering additionally for TFs with decreased abundance under acute ER stress (six total; potentially due to RESS), only *clr-2* and *xlr-1* overlapped, implying CLR-2 and XLR-1 maybe the only direct activators of the lignocellulase regulon. Trying to find the key repressor of cellulase genes, we reasoned that such a regulator might be up-regulated under ER stress (mild as well as acute) and down-regulated under standard cellulolytic induction (Avicel only) compared to the repression condition on sucrose. Only *cre-1* was identified to match these criteria (Figure 6B, lower panel) and is therefore confirmed to have a major influence on lignocellulase gene expression. However, this result does not imply that CRE-1 is the only repressor for cellulose genes, and additional repressors that are not necessarily co-regulated might still be involved. The expression pattern for *res-1*, *res-2*, and *rrg-2* did not correlate with major cellulase genes or core regulators such as *clr-2*, *xlr-1*, and *cre-1*, suggesting their influence on cellulase expression may occur through another way.

## Discussion

In the present study, we systematically analyzed the ER stress response of *N. crassa* during lignocellulase induction and secretion by experiments at both mild and acute stress levels. We found 766 genes to be specifically up-regulated in response to this process (ESRTs), of which 223 and 186 genes were regulated by UPR pathway core components IRE-1 and HAC-1, respectively. Screening 527 deletion strains for ESRTs lead to the identification of 249 genes that appear to be pivotal for resistance to ER stress in the WT. Of these, 100 targets were previously annotated as ‘unknown’ and are reported here for the first time to be involved in the response to ER stress. Interestingly, we found 357 ESRTs to be highly conserved, with orthologs in higher eukaryotes such as human (Additional file 3: Table S3 p3). For example, the ESRT NCU09101 encodes a hypothetical protein and has orthologs in both *S. cerevisiae* (YBR220C) and *H. sapiens* (SLC33A1/AT-1)*.* The function of *S. cerevisiae* YBR220C is unknown; however, *H. sapiens* SLC33A1/AT-1 encodes an ER membrane transporter that was recently reported to regulate the induction of autophagy downstream of the IRE1/XBP1 pathway [36]. Our screening data showed *ΔNCU09101* was sensitive to DTT (Additional file 5: Table S4), consistent with previous reports, validating that this target is related to ER stress. To our knowledge, systematic information on ER stress at a cellular level has only been performed in three model systems to date, including screening of KO mutants in *S. cerevisiae* [37] and screening of natural variation of human B cells [38] and *Drosophila* lines [39]. Thus, our data not only fill the knowledge gap in filamentous fungi but also contribute to the general understanding of the mechanisms mediating this process in higher eukaryotes.

In this study, we further addressed how TF networks coordinate the signal flow and gene expression during the ER stress response and cellulase synthesis. Thirty-three TFs were up-regulated upon ER stress under conditions of cellulase synthesis, including the well-characterized UPR regulator HAC-1 (NCU01856) as well as CPC-1 (NCU04050). We found *N. crassa* HAC-1 to act as an important factor for lignocellulase secretion while not mediating the RESS feed-back loop (profiling data mentioned above are presented in Additional file 1: Table S1 p2). In addition, we found that hyphal growth is severely impaired in *hac-1* deletion strains of *N. crassa*, which is consistent with previous observations in *Aspergillus niger* [40], suggesting that HAC-1 plays an important role in hyphal polarized growth and development, as it was shown in *Candida albicans* [28]. Interestingly, though the observed phenotype of Δ*ire-1* was slightly weaker than the phenotype changes in Δ*hac-1*, both mutants showed a pronounced defect in their conidial germination and cellulase secretion capacity compared to their parental strains. Moreover, an un-spliced *hac-1* transcript is present in the *N. crassa* Δ*ire-*1 strain. While in *S. cerevisiae* an un-spliced *hac-1* mRNA cannot be translated into a functional protein [41], previous studies could show that mammalian cells are indeed capable of translating un-spliced *hac-1/Xbp-1* transcripts [42]. Whether such an event also occurs in filamentous fungi and in which way these proteins would contribute to certain physiological functions such as hyphal polarized growth remain unclear. The TF CPC-1 has been previously characterized as a regulator associated with amino acid biosynthesis in *N. crassa* [43]. In metazoans, ATF4 as the functional homolog of CPC-1 has been proven to act in the PERK mediated UPR branch and it regulates those UPR genes involved in redox response and apoptosis [6,7,11]. The fact that CPC-1/CpcA is specifically induced upon ER stress has been observed in several independent fungal systems [11,44,45] and could be confirmed by our data. Here, we found additionally that a deletion of *cpc-1* renders *N. crassa* cells sensitive to DTT (Figure 5B) while significantly increasing extracellular protein secretion under limited nitrogen conditions (Figure S5). These observations suggest that CPC-1 may play a critical role in *N. crassa* ER stress response and cellulase secretion, although its regulation does not seem to be controlled by the IRE-1/HAC-1 mediated UPR cascade (Additional file 7: Table S5 p5). With NCU04058, a novel putative bZIP TF was identified, whose expression levels were markedly elevated upon acute ER stress. Moreover, the fact that the corresponding deletion strain displayed an apparent DTT-sensitive phenotype implies that it might also function as a stress response regulator.

The TF genes *res-1* (NCU03699) and NCU03043 are the homologs of *A. fumigatus zfpA* and *flbC*, respectively. It has been reported that both *A. fumigatus zfpA* and *flbC* transcripts can be induced by exposing cells to calcium [46,47], suggesting RES-1 and the TF encoded by NCU03043 may respond to intracellular calcium disturbances induced by ER stress. Moreover, we saw that loss of RES-1 results in a boost of lignocellulase secretion, probably owing to the de-repressive effect of several auxiliary cellulose-degrading enzymes on transcription (for example, several AA9 family members) which contribute to promote the efficiency of classical hydrolytic enzymes (cellulases). Deletion of *res-2* (NCU02724) and *rrg-2* (NCU02413) on the other hand, significantly decreased lignocellulase expression and secretion in *N. crassa*, although probably in an indirect manner. In this work, FunCat characterization of the RES-2 regulon revealed that genes involved in cellular export and secretion (*P* = 7.68e-03) as well as cellular import (endocytosis) (*P* = 5.49e-03) were enriched. Moreover, endocytosis has been reported to contribute to hyphal tip growth and tip secretion [48], suggesting RES-2 might regulate secretory pathways of *N. crassa*. In addition, the ortholog of RES-2 in *A. fumigates,* Afu1g09670, has been reported to be induced by calcium treatment [46]. While ER stress is always associated with a disturbance in the intracellular calcium homeostasis, changes in calcium levels are also reported to influence the hyphal tip growth [49,50] and thus might in turn affect tip secretion. Therefore, it is feasible, that RES-2 is involved in the ER stress response and affects lignocellulase secretion.

The *S. cerevisiae* SKN7, a homolog of the *N. crassa* RRG-2 (Identity: 57.5%, *E* value: 1E-38), controls the oxidative stress response in yeast cells together with YAP1 [51]. It has been reported that over-expression of *YAP1* contributes to modified intracellular redox conditions and enhances recombinant protein secretion [52]. However, whether SKN7 is also involved in protein secretion remains to be elucidated. In this work, deletion of RRG-2/SKN7 in *N. crassa* leads to a hypo-secretion phenotype, suggesting that RRG-2/SKN7 maybe has similar functions as YAP1 in the protein secretion process. It is also known that ER stress and oxidative stress are closely linked events, and therefore antioxidants, which can diminish oxidative stress [53], could additionally decrease ER stress and improve protein secretion. Hence, it can be concluded that RRG-2 might be involved in the regulation of the redox balance to affect cellulase secretion. The TF TAH-3 (NCU03686) was previously found to be required for fungal tolerance to harsh non-thermal plasma treatment in *N. crassa* [54]. The counterpart of TAH-3 in *S. cerevisiae* is UPC2, which functions by regulating sterol biosynthetic gene expression [55]. Potentially, disturbed sterol biosynthesis could trigger ER stress [56]. We found that extracellular protein secretion was reduced by circa 30% in *Δtah-3* compared to the WT, suggesting TAH-3 might be involved in ER stress resistance by maintaining intracellular sterol homeostasis.

The TF encoded by NCU09333 was identified as the homolog of the cellulase repressor ACE1 in *T. reesei* [57] and the stress responsive factor StzA in *Aspergillus nidulans* [58]. In *N. crassa*, NCU09333 was up-regulated upon ER stress while showing only a limited effect on cellulase secretion, suggesting NCU09333 functions more like StzA rather than ACE1 in *N. crassa*. Finally, the expression level of the carbon catabolite repression (CCR) regulator CRE-1 (NCU08807) was found to be up-regulated upon ER stress. The *Δcre-1* strain moreover showed a DTT-sensitive phenotype, suggesting the CCR pathway might also cross-talk with the ER stress response.

**Figure 7:**
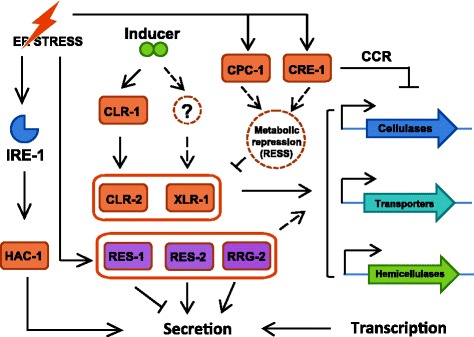
Schematic model illustrating the coordinated action of ER stress and cellulase synthesis. ER stress affects cellulase gene expression and secretion via the core lignocellulase regulators. Acute ER stress specifically down-regulates *clr-2* and *xlr-1* rather than *clr-1* for rapid adjustment of lignocellulase transcript abundance. The CRE-1-mediated CCR pathway activated by ER stress might also contribute to this process. The newly identified regulators RES-1, RES-2, and RRG-2 indirectly affect lignocellulase production. However, enzyme synthesis was increased in *Δres-1* when compared with WT, whereas *Δres-2* and *Δrrg-2* showed repressive effects, suggesting RES-1 likely acts as repressor while RES-2 and RRG-2 act as activators for the synthesis and secretion of extracellular enzymes. The IRE-1/HAC-1-mediated UPR pathway also acts indirectly on lignocellulase production by mainly regulating protein folding, modification (such as glycosylation), and transport. The finding of RESS being independent of UPR pathway, raise the possibility that metabolic repression resulting from ER stress might contribute to this process.

By monitoring TFs with a decreased abundance upon ER stress, we detected CLR-2 and XLR-1. This result was confirmed by qPCR (Additional file 4: Figure S8), suggesting that the RESS-mediated repression of cellulase genes was likely a direct result of the down-regulation of these two regulators. The expression levels of some carbohydrate transporter genes, which are belonging to the *clr-2* and *xlr-1* regulons, such as *cdt-1* (NCU00801) and *lat-1* (NCU02188), were also decreased, supporting our hypothesis. It was intriguing to find that the transcription level of another key regulator, cellulose degradation regulator 1 (CLR-1), was not significantly down-regulated in the RESS condition (Additional file 4: Figure S8). CLR-1 has been shown to be necessary for expression of *clr-2* [33] and is up-regulated when fungal cells are exposed to cellulose, suggesting the expression of *clr-1* may be directly regulated by biopolymer inducers while *clr-2* transcription is not only controlled by CLR-1 but also regulated by some other players in the ER stress response. With the discovery mentioned above, a new model for the lignocellulase induction and synthesis of *N. crassa* is presented (Figure 7).

RESS was shown to be activated independently of the IRE-1/HAC-1 mediated UPR pathway in this work (profiling data on Additional file 1: Table S1 p2 and Figure 7). How RESS is triggered by ER stress remains to be elucidated. Notably, a previous study in *T. reesei* demonstrated that the intracellular concentrations of some free amino acids increased upon acute ER stress [44]. Moreover, we found *cre-1* expression levels to be elevated when cells suffered ER stress, suggesting that the intracellular glucose homeostasis may also be perturbed under these conditions. These observations raise the possibility that metabolic repression that results from internal fluctuations of intracellular nutritional cues, such as simple sugars or free amino acids, might have an impact on RESS, although more data need to be acquired to support this hypothesis.

## Conclusions

Here, we deeply investigated the complicated cross-talk between the ER stress response and lignocellulase synthesis in *N. crassa* by combining transcriptional profiling, mutant screening, and a detailed regulatory network analysis. The genome-wide screening uncovered hundreds of genes that might be involved in this pathway, including 100 formerly hypothetical genes. Our data furthermore allowed connecting the function of three novel transcription factors to cellulase expression and the ER stress response, shedding new light on the molecular mechanism of this complicated cross-talk in fungi. Since filamentous fungi are important systems for biotechnological enzyme production, the findings of the present study should have an impact on industrial fungal strain development for both lignocellulase production as well as synthesis of biomass-based chemicals.

## Materials and methods

### Strains

The *N. crassa* strains used in the study were obtained from the Fungal Genetics Stock Center (http://www.fgsc.net/) [59], including the WT strain FGSC#2489 (*Mat A*), FGSC#9720 (*mus-52::bar; his-3, Mat A*), the *cpc-1* mutant (CD15 allele mutation, FGSC#4264) and a set of 526 homokaryotic deletion strains individually knocked-out in non-essential genes encoding proteins playing potential roles in ER stress response. The *hac-1* gene was deleted following standard methods provided by the *Neurospora* Functional Genomics Project (http://www.fgsc.net/ neurosporaprotocols/KO_Protocols.pdf, for details see Additional file 6: Supporting Information), while a homokaryotic *ire-1* KO mutant was generated by a sexual cross of the heterkaryon FGSC#21727 with wild-type strain FGSC#2489.

### Liquid cultures for ER stress stimulation

For strains able to utilize cellulose, such as WT, *Δres-1*, *Δres-2*, and *Δrrg-2*, liquid cultivations were carried out on a defined cellulose medium (1× Vogel’s salts, 1% *w*/*v* crystalline cellulose (Avicel PH-101; Sigma-Aldrich, St. Louis, USA), 0.2% *w*/*v* NH_4_NO_3_) according to published data [60]. Ten-day-old conidia were inoculated into a 50-mL medium at 10^6^ conidia/mL final concentrations and grown at 25°C in constant light and agitation (200 rpm). After 36 h, the cultures were treated with either dithiothreitol (DTT) or tunicamycin (TM) with indicated doses for one more hour to induce ER stress. Since both strains, *Δire-1 and Δhac-1*, are unable to utilize cellulose, both mutants and their parental strains were pre-grown in minimal medium (1× Vogel’s salts, 2% *w*/*v* sucrose) for 16 h, and then young hyphae were harvested and transferred into cellulose medium as mentioned above for another 4 h to induce cellulase gene expression. After that, 5 mM DTT were added into each culture to stimulate ER stress for one more hour.

### ER stress sensitivity screening

Fresh conidia were harvested from 10-day-old slants for each strain and suspended in sterilized water to a final concentration around 3 × 10^5^ per mL. Three microliters of a conidia suspension (approximately 1,000 conidia) were spotted onto modified solid GFS medium [61] (1× Vogel’s salts, 2% *w*/*v* sorbose, 0.05% *w*/*v* glucose, 0.05% *w*/*v* fructose, 1.5% *w*/*v* agar) including the indicated dose of DTT or TM. Colonial growth was performed at 25°C for 3 days. The antibiotic Hygromycin B was added into the medium at a final concentration of 200 μg/mL for all knock-out mutants, while L-histidine was added with a final concentration of 100 μg/mL for *Δhac-1* and FGSC#9720.

### Total RNA extraction and real-time qPCR analysis

Total RNA from frozen samples was isolated using Zirconia/Silica beads (0.5 mm diameter; Biospec Products, Bartlesville, USA) and a Mini-Beadbeater (Biospec Products, Bartlesville, USA) with 1 mL TRIzol reagent (Invitrogen, Grand Island, USA) according to the manufacturer’s protocol. An additional clean-up including the on-column DNase I treatment was performed by using the RNeasy mini kit (QIAGEN, Valencia, USA). For qPCR analysis, reverse transcription was performed using iScript™ cDNA synthesis kit (Bio-Rad, Hercules, USA). The qPCR was performed using iQ™ SYBR Green Supermix (Bio-Rad, Hercules, USA) on the CFX connect™ Real-Time system (Bio-Rad, Hercules, USA). PCR reaction setup was according to the manufacturer’s instructions. The relative transcript level of each gene was calculated by the 2-ΔΔCt (comparative Ct) method. Data were normalized to the endogenous control *actin* (NCU04173) with expression on untreated control as the reference sample.

### RNA sequencing and data analysis

The total RNA of two biological replicate samples was extracted separately, mixed together after measuring the quality of each sample, and used for high-throughput RNA sequencing. The 13 49-nt single-end RNA-seq libraries were generated commercially at Beijing Genomics Institute (BGI, Shenzhen, China) while eight 90-nt paired-end RNA-seq libraries were generated at GENEWIZ (Biotechnology Co. Ltd, Suzhou, China) by using Illumina’s HiSeq™ 2,000 platform (Illumina, San Diego, USA) (see summary of Additional file 1: Table S1 for details). Most of the data in the present study were generated by sequencing only one sample. However, as a reference, we performed parallel sequencing in this study for WT to make sure the sequencing data we got were technically reliable. The data of libraries 01 and 14 (Additional file 1: Table S1) come from biological replicates (WT no drug control), similar as the data from libraries 03 and 15 (Additional file 1: Table S1, WT treated with 5 mM DTT). The correlations between the two RNA-seq replicate data were analyzed and are shown in Additional file 4: Figure S9.

The sequencing data are deposited in the Gene Expression Omnibus database (GEO, http://www.ncbi.nlm.nih.gov/geo/) with accession number GSE61949. Filtered clean eads were then aligned to the latest *N. crassa* OR74A genome (version 12) [62] (http://www.broadinstitute.org/annotation/genome/neurospora/MultiHome.html) with splicing-aware aligner TopHat2 (version 2.0.12) [63]. Abundance for each transcript was calculated using the reads per kilobase per million (RPKM) [64]. Genes with altered expression was performed by using R package NOISeq (version 2.6.0) [65] (*Q* value ≥0.95 or 0.90 used as threshold for single-end or paired-end libraries, respectively, which approximately corresponds to a |log_2_ ratio| ≥1). To discover significantly expression changes between the conditions tested, only the genes with relative high transcriptional abundance (RPKM values above 20 or 15 in at least one condition for single-end or paired-end libraries, respectively [66]) were deemed as positives and went into further analysis. MIPS FunCat online tools (http://mips.helmholtz-muenchen.de/funcatDB/) were used to functional classification of genes with altered expression. Meanwhile, manual annotation was assigned to these genes by referring to Blast hits and Pfam domain annotation (Broad version 7) to prove or verify FunCat results (for detail, see Additional file 6: Supporting Information).

### Growth, lignocellulase secretion, and enzyme activity assays

Liquid cultures for cellulase production were carried out either in cellulose medium with rich nitrogen source (1× Vogel’s salts, 2% *w*/*v* Avicel, 0.75% *w*/*v* yeast extract) or poor nitrogen source (1× Vogel’s salts, 2% *w*/*v* Avicel). The 10-day-old conidia of tested strains were inoculated into 100 mL liquid media at 10^6^ conidia/mL final concentration and grown at 25°C in constant light and shaking (200 rpm) over the course of 7 days. Since the deletion strain from the TF NCU03043 had severe defects in conidiation, young hyphae were pre-grown in minimal medium for 24 h to accumulate biomass, which was then shifted into rich/poor nitrogen source medium and fermented for 6 days to produce lignocellulases. For switching experiments with the hyper- or hypo-secretion mutants (such as Δ*res-1*, Δ*res-2*, and Δ*rrg-2*), 10-day-old conidia were inoculated into 100 mL minimal medium at 10^6^ conidia/mL final concentration and grown at 25°C in constant light and agitation (200 rpm). After 16 h, young hyphae were harvested and transferred into cellulose medium with rich nitrogen source for the indicated times to produce cellulases. Relative enzyme activity of culture supernatants was assayed with Remazol brilliant Blue R-conjugated CMC kits (Megazyme, Wicklow, Ireland). The amount of secreted protein was determined using the Bradford protein assay (Bio-Rad, Hercules, USA). For protein gel electrophoresis, 4-μl unconcentrated culture supernatants were loaded on SDS-PAGE (Novex® NuPAGE® Pre-cast Protein Gels, Thermo fisher scientific, Waltham, USA). Directly measuring the dry weight of fungal biomass was replaced by measuring the total protein content in order to mitigate the interference of Avicel on the test. Assays were performed as previously described [67]. To check the actual protein loading of the secretory pathway of certain mutants (for example, Δ*hac-1*, Δ*ire-1*, Δ*res-1*, Δ*res-2*, and Δ*rrg-2*), total protein from the same amount of biomass for each strain grown on sucrose or Avicel were extracted as previously described [67]. Unconcentrated extractions (4 μl) for each sample were loaded on SDS-PAGE.

### Microscopy and image processing

The growth phenotype of Δ*ire-1*/Δ*hac-1* as well as their parental strains was monitored by inoculating around 1,000 conidia for each strain into the center of petri dishes containing minimal medium and growth for 3 days. For recording conidial and mycelial differential interference contrast (DIC) images, cultures were performed in liquid minimal medium and samples collected at indicated time points. DIC images were recorded by using an Olympus BX51 Microscope (Olympus, Tokyo, Japan). Image Pro Plus (v6.0) was used for image processing.

### Statistical significance tests and data plotting

Unless otherwise noted, all experiments were performed in triplicate and statistical tests for significance determined via one-way ANOVA by using R (version 3.1.1). R Packages include pheatmap (version 0.7.7) [68], ggplot2 (version 0.9.3.1) [69], VennDiagram (version 1.6.9) [70], and phenotypicForest (version 0.2) [71] used to data plotting.

## Additional files

Additional file 1: Table S1. RNA-seq read profiles mapped to the genome of *N. crassa*. Additional file 2: Table S2. Differential expression analysis. Additional file 3: Table S3. Functional groups of genes with altered expression in response to ER stress with different stress intensities. Additional file 4: Figures S1-S9. Supplementary figures referred to in the main text. Additional file 5: Table S4. Phenotypical screen of 527 ESRT KO mutants under DTT-induced ER stress. Additional file 6:
**Supporting information.** Additional methods referred to in the main text. Additional file 7: Table S5. Genes regulated by either IRE-1 or HAC-1 in *N. crassa.*
Additional file 8: Table S6. TFs associated with ER stress response and cellulase synthesis in *N. crassa.*
Additional file 9: Table S7. Genes regulated by the TFs RES-1, RES-2, or RRG-2 in *N. crassa*.

## Abbreviations

Bp: base pair
*clr-1*/*clr-2*: cellulose degradation regulator 1/2
*cpc-1*: cross pathway control protein 1
ER: endoplasmic reticulum
ESRTs: ER stress response targets
*hac-1*: homologous to ATF/CREB1
*ire-1*: inositol-requiring enzyme-1
Nt: nucleotide
ORF: open reading frame
*res-1/res-2*: regulator for ER stress response 1/2
RESS: repression under secretion stress
RPKM: reads per kilobase per million
*rrg-2*: response regulator 2
TF: transcription factor
UPR: unfolded protein response
*xlr-1*: xylan degradation regulator 1
