# Supplementary material for: Genome-wide analysis of the endoplasmic reticulum stress response during lignocellulase production in Neurospora crassa
Source: Biotechnol Biofuels. 2015 Apr 14;8:66. doi: 10.1186/s13068-015-0248-5 (PMC4399147; doi:10.1186/s13068-015-0248-5)
Supplement: Additional file 4: Figures S1-S9. — Supplementary figures referred to in the main text. [file 13068_2015_248_MOESM4_ESM.pdf]

Supplemental Figures S1-S9

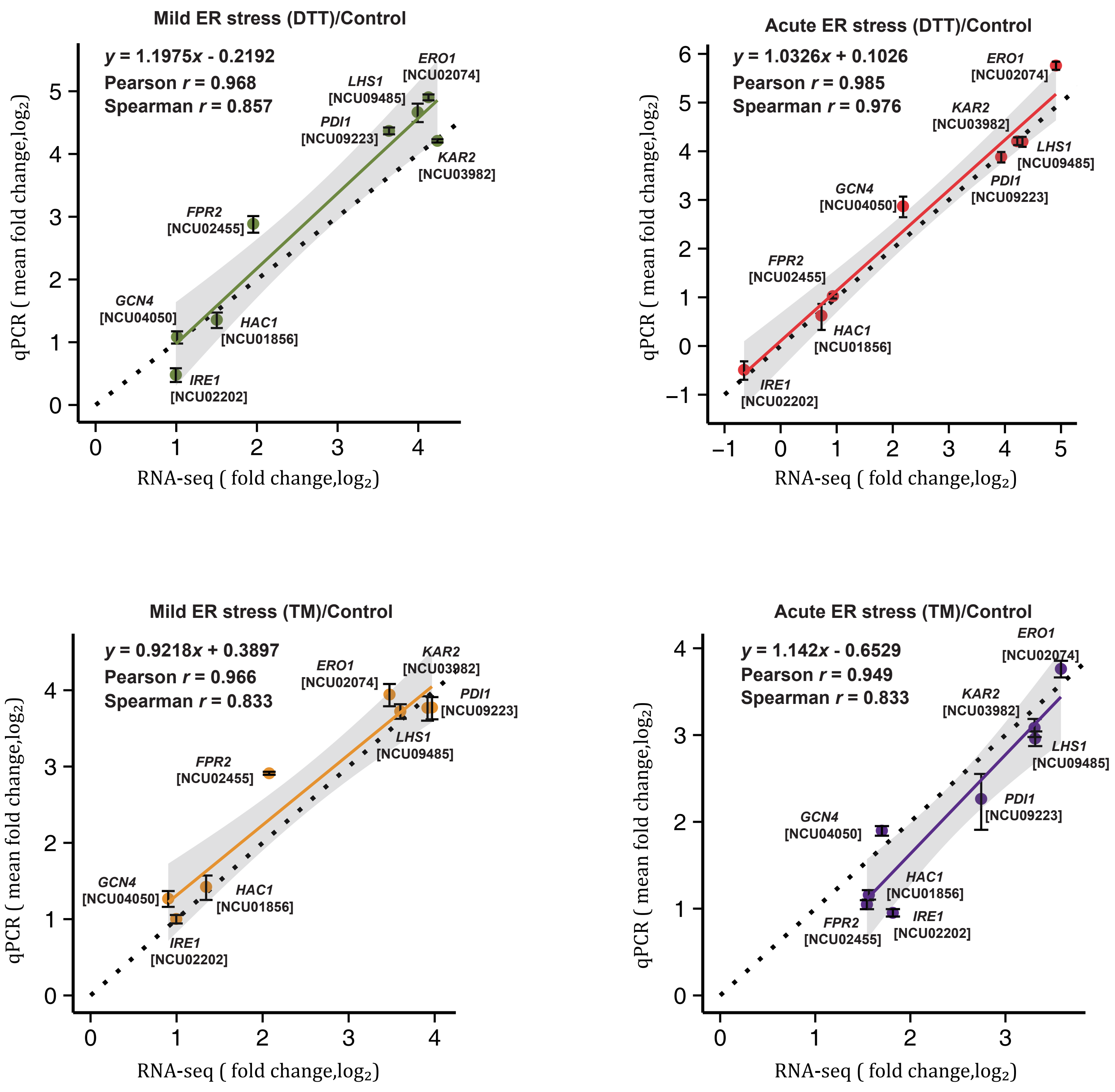

Figure S1. Correlation of RNA-seq and qPCR assay data. RPKM values for RNA-seq data and mean normalized fold change for qPCR data, presented with their respective standard error of the mean (SEM, n=3), are matched against each other. The linear regression line (colored) and the identity line (black, dotted) are shown in each panel. The 95% conffidence interval of each regression line is represented as grey-shaded area. Pearson's as well as Spearman's r-value were calculated and show good correlation between the independent results. The r-values and the linear equation of each regression line are presented in the top left corner of each diagram.

A

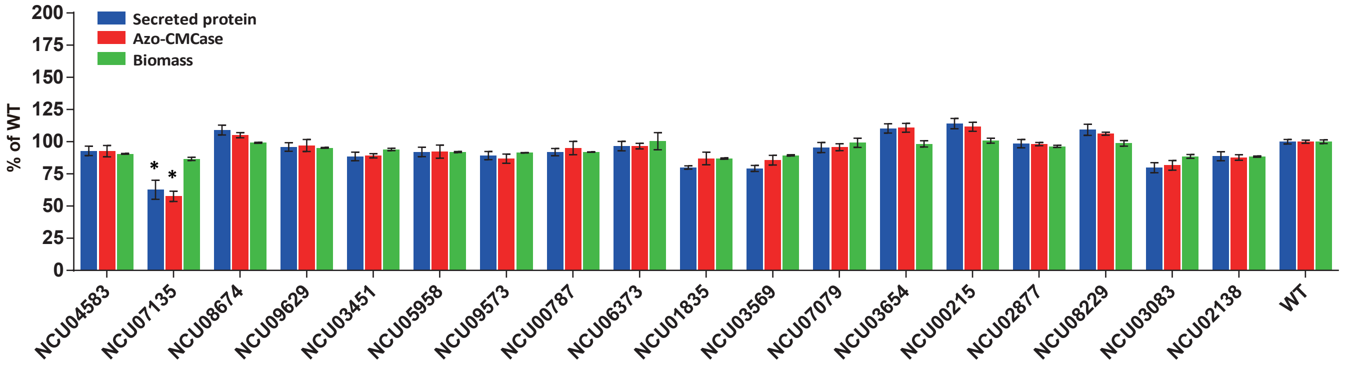

B

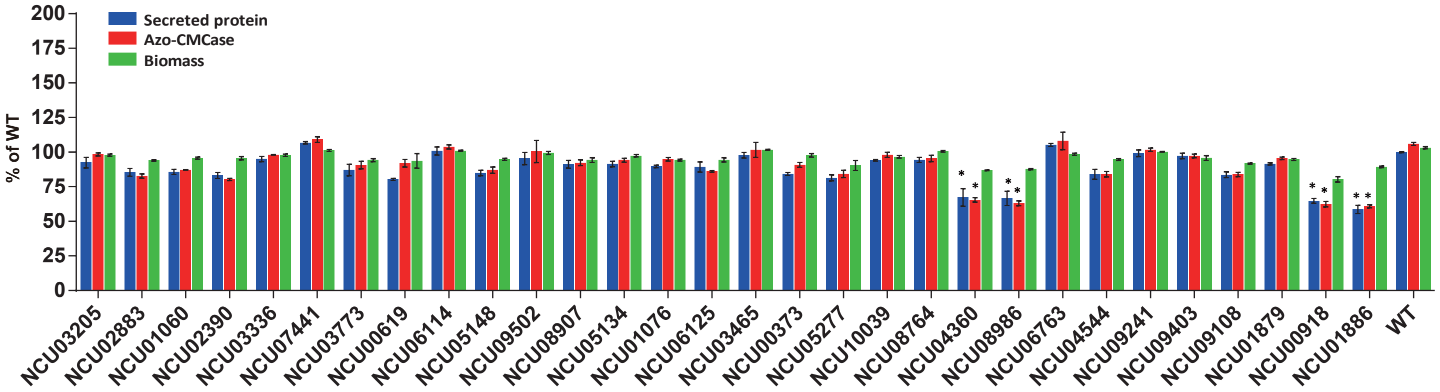

Figure S2. Cellulase production of KO mutants of ESRTs with unknown function. The strains which express hypothetical proteins that are sensitive to 3 mM DTT (A) or 5 mM DTT (B) were tested. After growth for 7 days on Avicel with additional 0.75% yeast extract as rich nitrogen source, the extracellular protein concentration, CMCase activity and biomass of the mutant strains was measured and compared to WT (FGSC# 2489). The values of the WT strain were used as 100% for comparison with the mutant strains. All values are presented with their Standard error of the mean (SEM, n=3). Significant changes were calculated by using a one-way ANOVA, the asterisks (*) indicate a significant difference from the WT (unadjusted P-value of < 0.001).

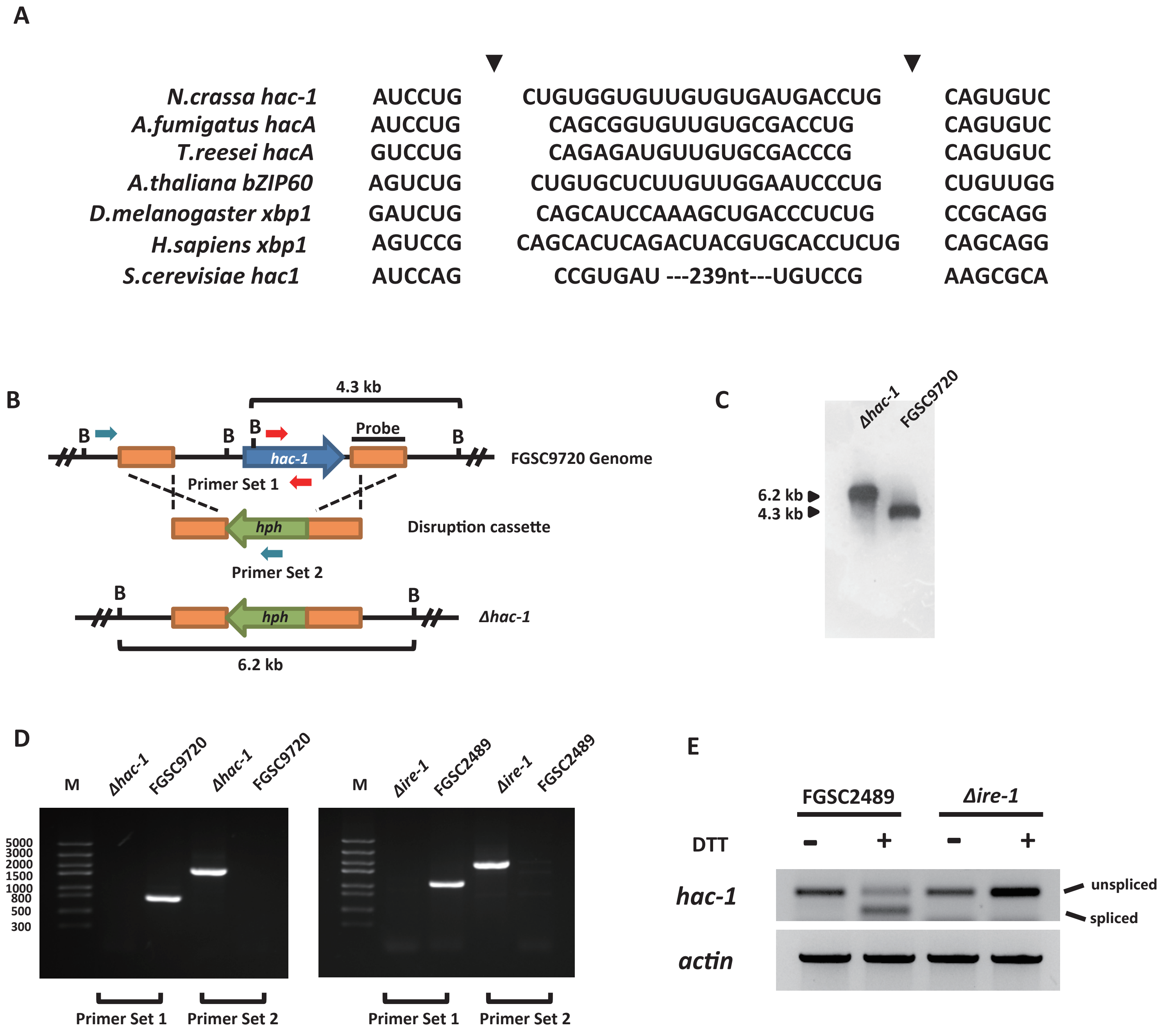

Figure S3. (A) Length comparison of the unconventionally spliced *hac-1* intron in different species. A close phylogenetic link between higher eukaryotes and filamentous fungi was revealed by comparing the length of the unconventionally spliced *hac-1* intron of *N. crassa* with introns of *hac-1* orthologs in other species. (B) Schematic overview of the *hac-1* deletion by using homologous recombination. The restriction enzyme *BglII* is represented by a capital B in the images, and this enzyme was used for digesting the genomic DNA for southern blotting. The 3' flank region was used as probe. (C) Southern blot result. (D) Agarose gel of the PCR which was used to confirm that the *ire-1* and *hac-1* KO mutants are homokaryotic. (E) The IRE-1 mediated unconventional splicing of *hac-1* mRNA in *N. crassa*. WT and *Δire-1* were pre-grown on minimal medium for 16 h and afterwards treated with 5 mM DTT for 1 hour (+). The control was not treated with DTT (-). RT-PCR was used to reveal the alternative splicing of *hac-1*.

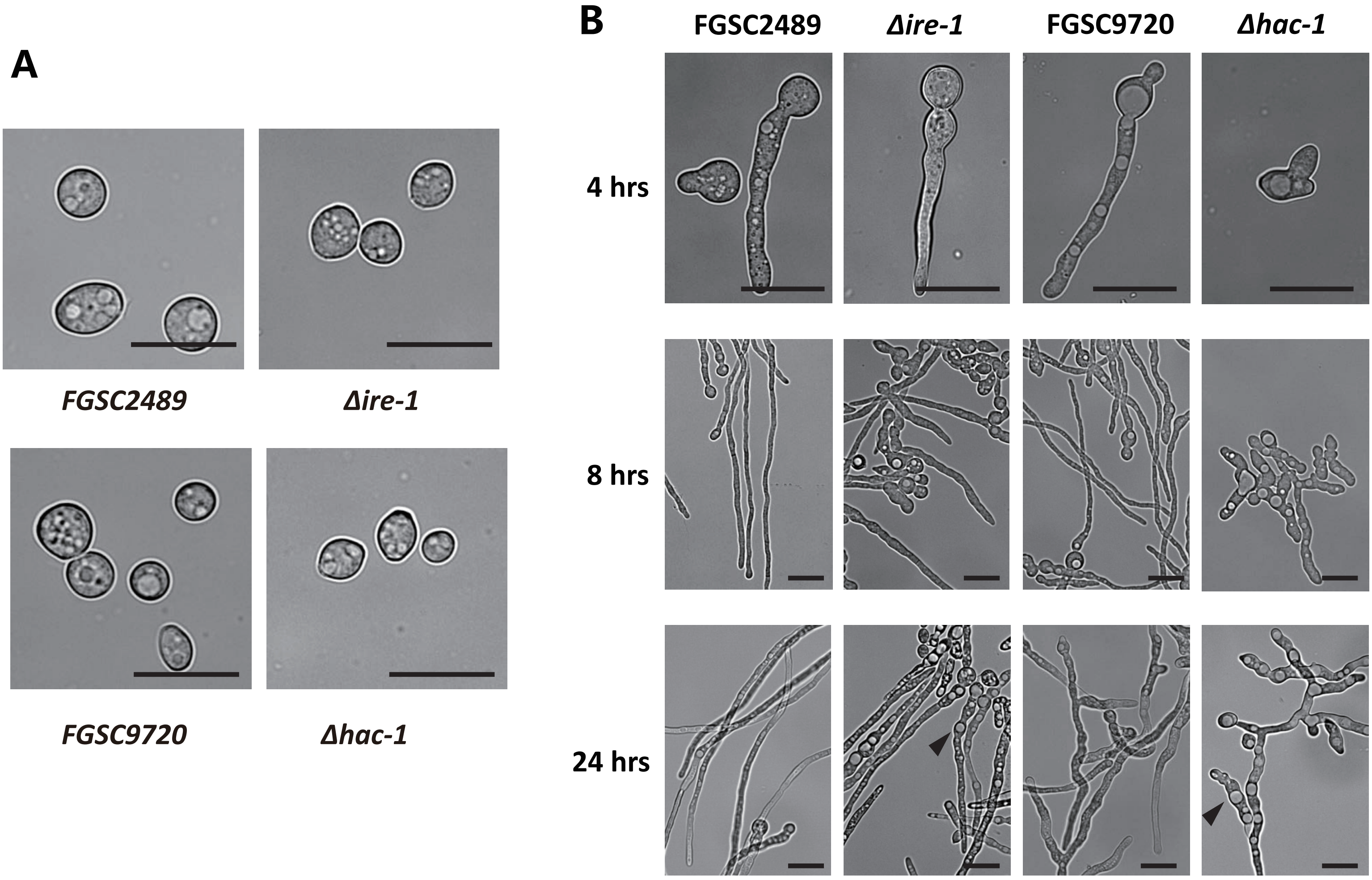

Figure S4. Morphological analysis of Δ*ire-1* and Δ*hac-1*. (A) Typical conidial morphology for Δ*ire-1* and Δ*hac-1* as well as their parental strains. Bars = 10 μm. (B) Germination morphology in minimal medium. Aberrant vacuoles accumulated within the Δ*ire-1* and Δ*hac-1* mutants (black arrow). Bars = 10 μm.

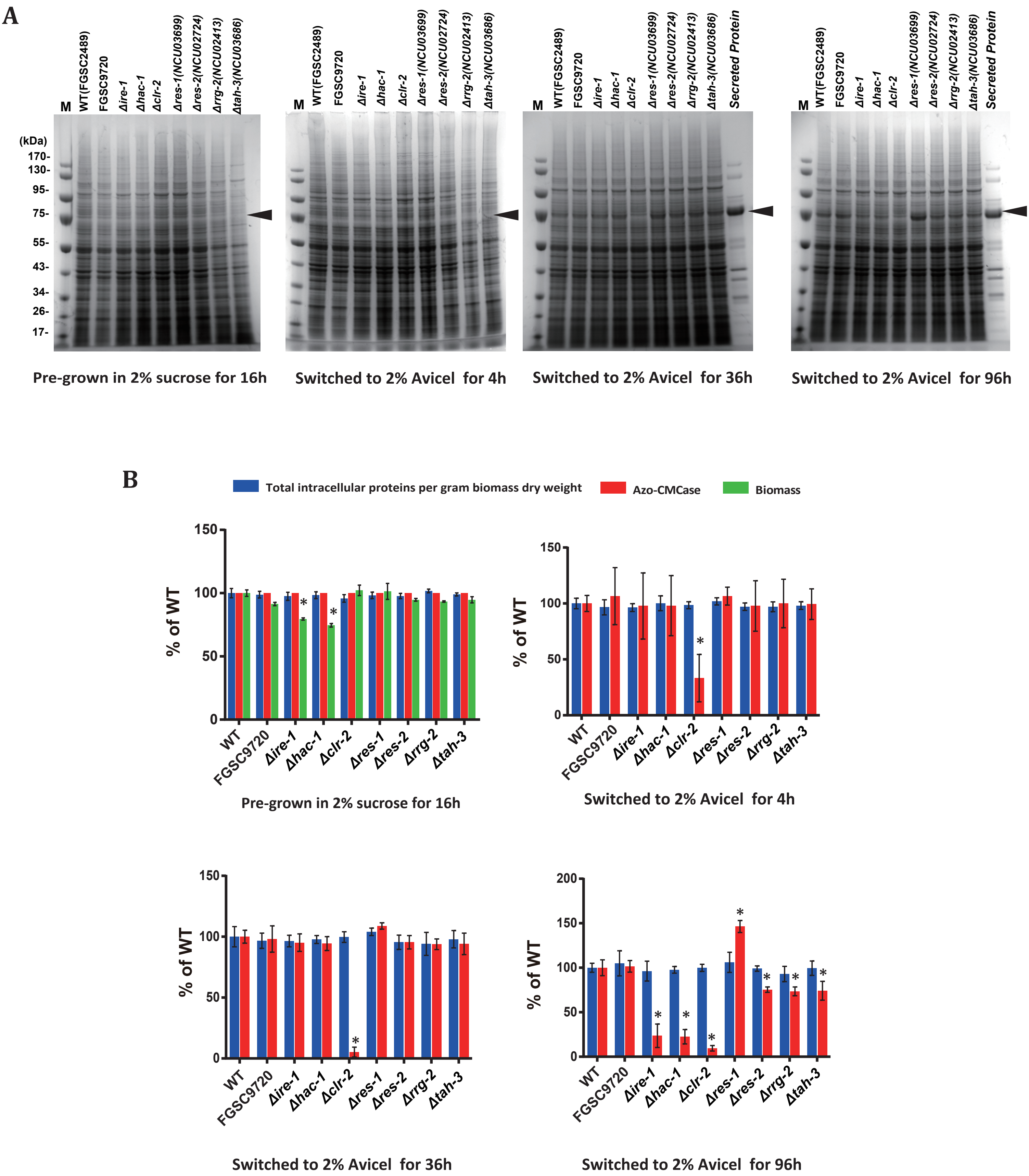

Figure S5. Examination of the intracellular cellulase translation and actual protein load of the secretory pathway of hyper-/hypo-secretion *N. crassa* mutants. (A) SDS-PAGE of total intracellular protein of six hypo-secretion mutants (including Δ*ire-1*, Δ*hac-1*, Δ*clr-2*, Δ*res-2*, Δ*rrg-2* and Δ*tah-3*), one hyper-secretion mutant (Δ*res-1*) as well as two control strains (WT FGSC# 2489 and FGSC# 9720). All strains were grown on sucrose for 16 h and then switched to Avicel containing medium (using the same amount of biomass for all samples) for another 4 h, 36 h or 96 h. The position of the typical cellulases in a WT strain (e.g. CBH-1 and CBH-2) are shown with black arrows on the profile of the extracellular secreted proteins on Avicel. (B) Measurement of total intracellular protein concentration per gram biomass dry weight, CMCase activity and biomass (corresponding to A). The data of all strains was compared to WT, which was set as 100%. All values are presented with their Standard error of the mean (SEM, n=3). Significant changes were calculated by using a one-way ANOVA; the asterisks (*) indicate a significant difference from the WT (unadjusted P-value of < 0.001).

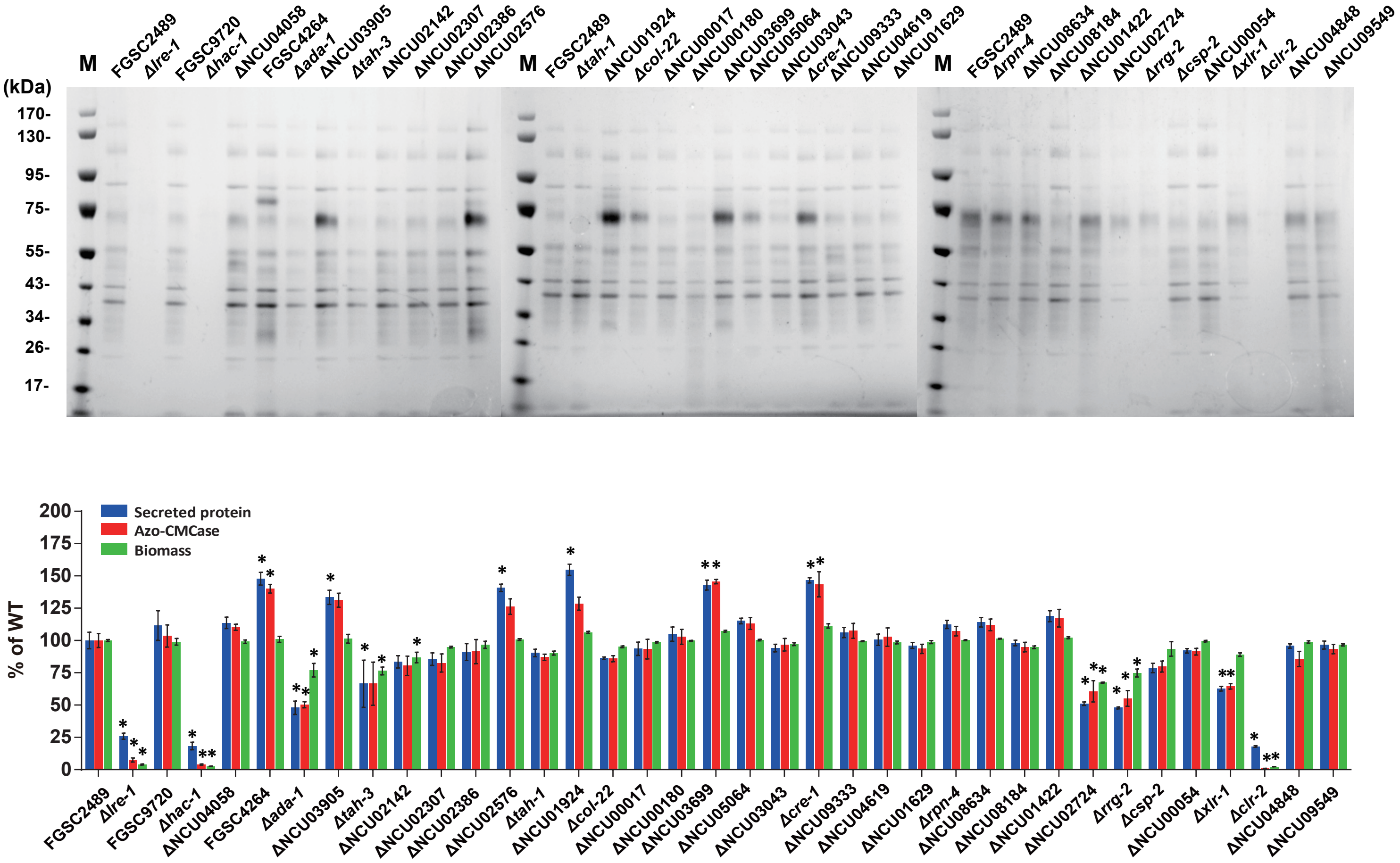

Figure S6. Cellulase production of 35 TF deletion strains. (Upper panel) SDS/PAGE of proteins present in the culture filtrates of 34 TF KO strains as well as Δ*cpc-1* (FGSC# 4264) compared to WT when grown on Avicel medium without additional rich nitrogen source for 7 days. (Lower panel) Extracellular protein concentration, CMCase activity and biomass accumulation were measured and compared with WT (FGSC# 2489), which was set to 100%. All values are presented with their Standard error of the mean (SEM, n=3). Significant changes were calculated by using a one-way ANOVA, the asterisks (*) indicate a significant difference from the WT (unadjusted P-value of < 0.001).

A

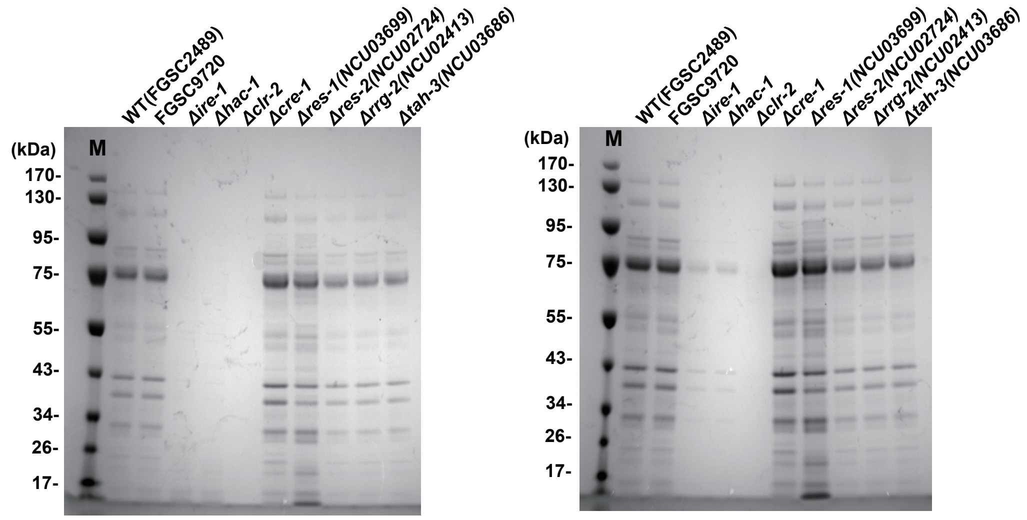

Pre-grown in 2% sucrose for 16h then switched to 2% Avicel for 36h

Pre-grown in 2% sucrose for 16h then switched to 2% Avicel for 96h

B

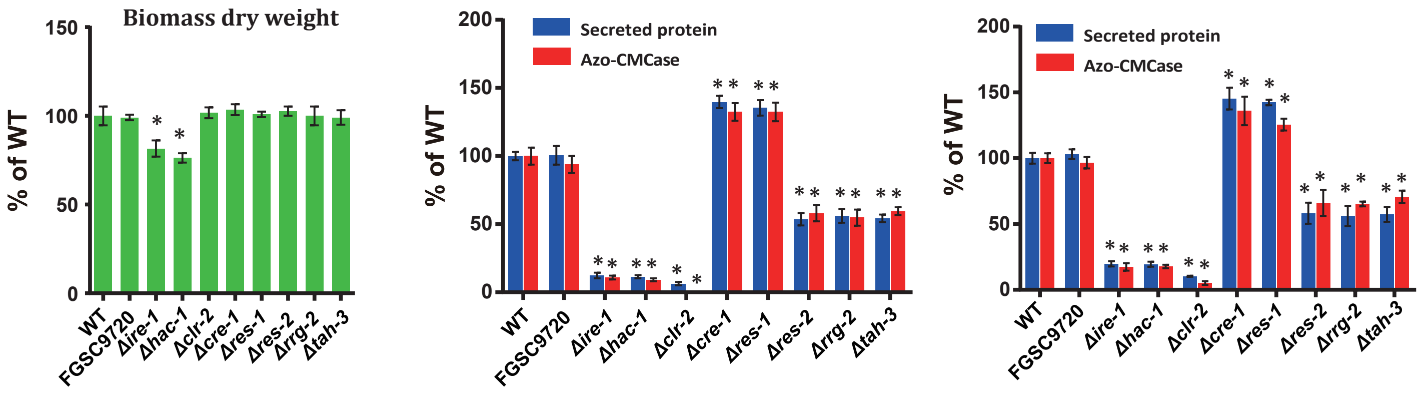

Pre-grown in 2% sucrose for 16h

Switched to 2% Avicel for 36h

Switched to 2% Avicel for 96h

Figure S7. Test of cellulase secretion in hyper-/ hypo-secretion mutants of *N.crassa* after carbon source switch. (A) SDS-PAGE of secreted proteins in culture filtrates from one hyper-secretion mutant (Δ*res-1*) as well as five hypo-secretion mutants (Δ*ire-1*, Δ*hac-1*, Δ*res-2*, Δ*rrg-2* and Δ*tah-3*) pre-grown on sucrose for 16h and then switched to Avicel medium (0.75% Yeast Extract) for another 36h and 96h, respectively. The WT (FGSC# 2489), FGSC# 9720 (Δ*mus-52*::*bar*+; *his-3*), Δ*clr-2* and Δ*cre-1* were included as controls. All strains were adjusted to the same amount of biomass before switch to cellulose utilizing conditions. (B) Biomass dry weight was determined after pre-growth on sucrose for 16h (left panel). Extracellular protein concentration and CMCase activity were determined after 36h and 96, respectively (same samples as in A; middle and right panel). All data are normalized to WT, which was set to 100%, and are presented with their Standard error of the mean (SEM, n=3). Significant changes were calculated by using a one-way ANOVA, the asterisks (*) indicate a significant difference from the WT (unadjusted P-value of < 0.001).

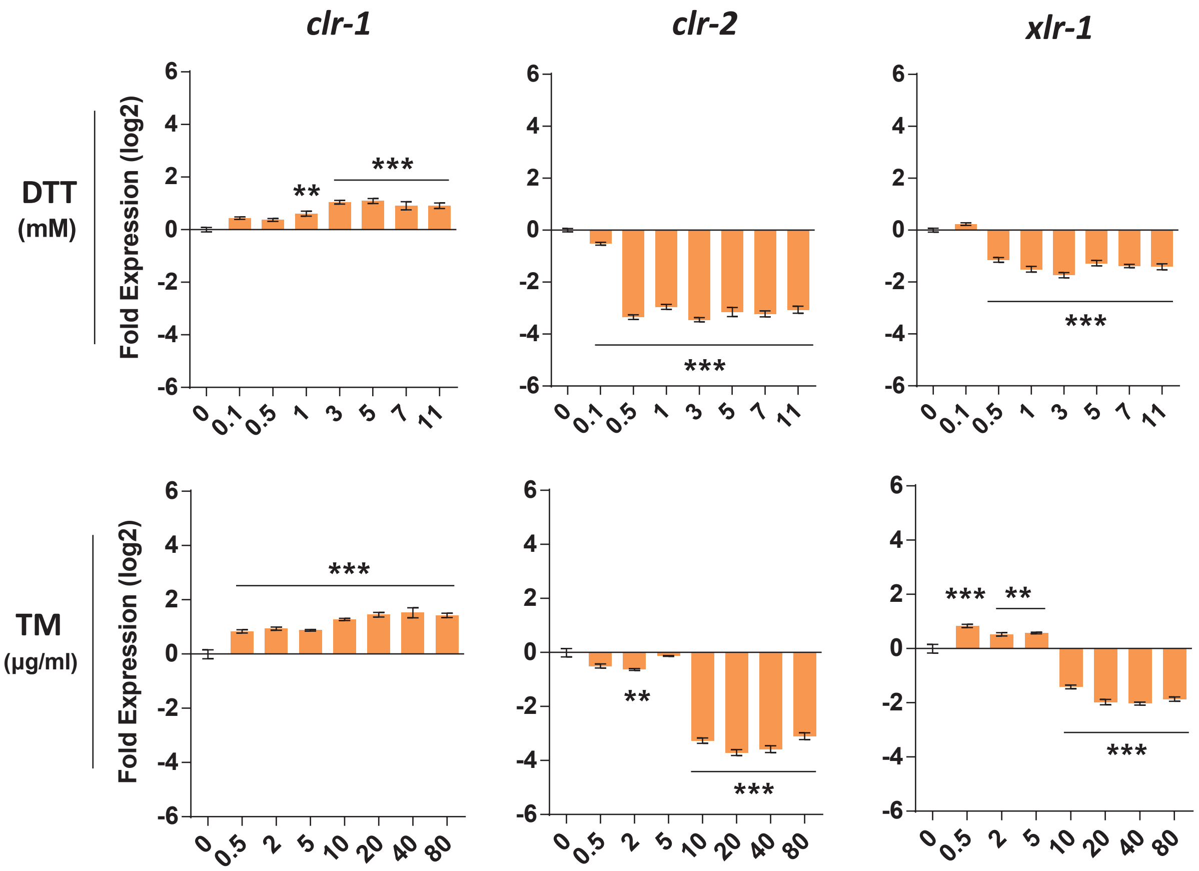

Figure S8. The TFs *clr-2* and *xlr-1*, but not *clr-1*, are coordinately downregulated with lignocellulase genes upon ER stress. Gene expression levels of core cellulase regulators under increasing ER stress levels induced by DTT (0.1 – 11 mM) and TM (0.5 – 80 µg/ml) were recorded by qPCR. The data are normalized to expression on mock = 1 and actin (NCU04173) gene expression levels were used as an endogenous control in all samples (standard error of the mean (SEM), n=3, the asterisk indicates a significant difference from mock (P < 0.01, ***P < 0.001) via one-way ANOVA).**

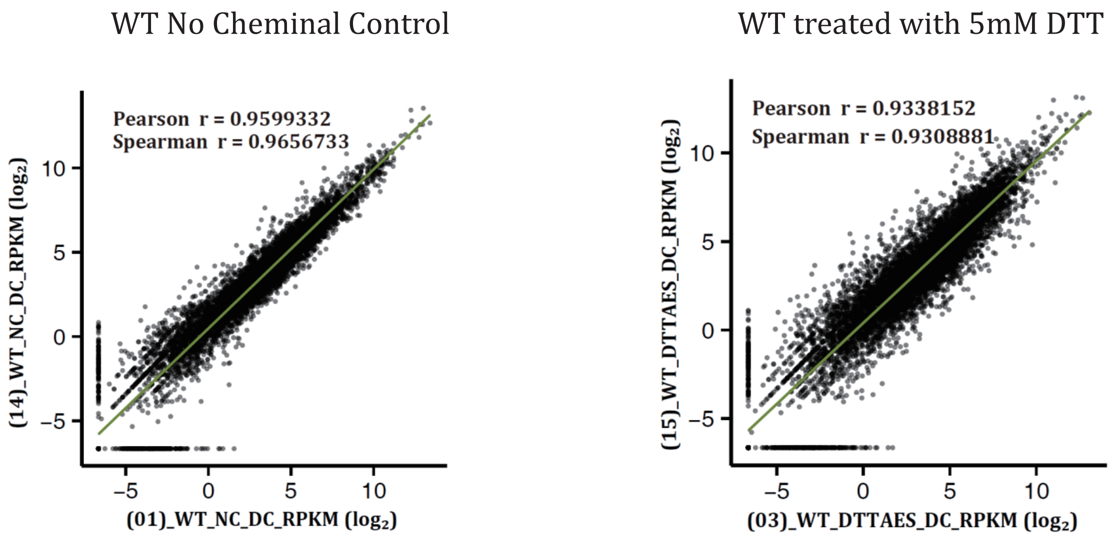

Figure S9. Inter-sample correlation comparison of RNA-seq data. The recorded RPKM values of both biological replicate RNA-seq libraries for "WT no chemical control" and "WT treated with 5mM DTT" were pairwise compared. The green line represents the linear regression line. Pearson's and Spearman's correlation coefficient were calculated and show strong correlation between the independent samples.
