# Supplementary material for: Genome-wide analysis of the endoplasmic reticulum stress response during lignocellulase production in Neurospora crassa
Source: Biotechnol Biofuels. 2015 Apr 14;8:66. doi: 10.1186/s13068-015-0248-5 (PMC4399147; doi:10.1186/s13068-015-0248-5)
Supplement: Additional file 5: Table S4. — Phenotypical screen of 527 ESRT KO mutants under DTT-induced ER stress. [file 13068_2015_248_MOESM5_ESM.pdf]

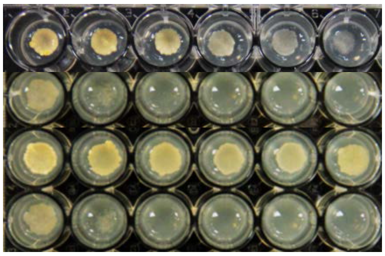

No.	Locus	Gene Product Names	Log2 ratio of RPKM (Median value, DTT&TM)	STDEV
	FGSC2489	*N. crassa* wild type A	Positive Control	
	NCU02202	serine/threonine kinase IRE-1	Positive Control	
	FGSC9720	delta mus-52::bar+; his-3 A	Positive Control	
	NCU01856	transcriptional activator HAC-1		

Mild Stress Specific Targets

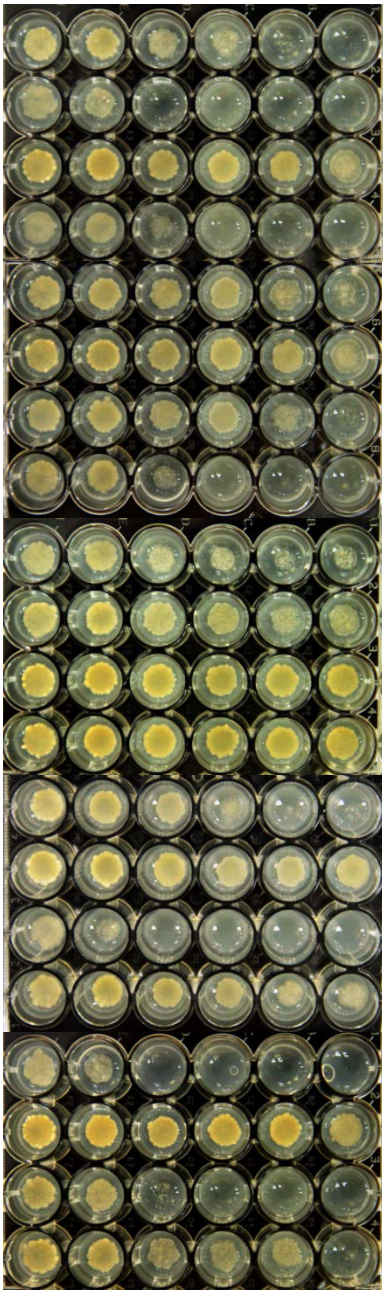

No.	Locus	Gene Product Names	Log2 ratio of RPKM (Median value, DTT&TM)	STDEV
001	NCU10538	hypothetical protein	4.99	1.56
002	NCU00787	hypothetical protein	4.55	0.44
003	NCU04197	CipC protein	4.29	0.66
004	NCU08907	BYS1 domain-containing protein	4.07	2.9
005	NCU03202	hypothetical protein	3.98	0.48
006	NCU00716	non-anchored cell wall protein 5	3.69	1.1
007	NCU07787	clock controlled protein CCG-14	3.57	2.05
008	NCU07129	amino-acid permease inda1	3.56	0.4
009	NCU06255	hypothetical protein	3.43	0.22
010	NCU04205	acid proteinase	3.19	0.19
011	NCU09471	hypothetical protein	3.19	0.17
012	NCU09470	hypothetical protein	3.12	0.23
013	NCU08490	hypothetical protein	3.03	0.71
014	NCU08489	transporter SMF1/ESP1	3.00	1.01
015	NCU05852	glucuronan lyase A	2.92	1.46
016	NCU07823	scytalone dehydratase	2.86	0.31
017	NCU09771	DUF895 domain membrane protein	2.85	0.27
018	NCU09472	hypothetical protein	2.77	0.05
019	NCU06373	hypothetical protein	2.74	0.3
020	NCU09224	hypothetical protein	2.72	0.35

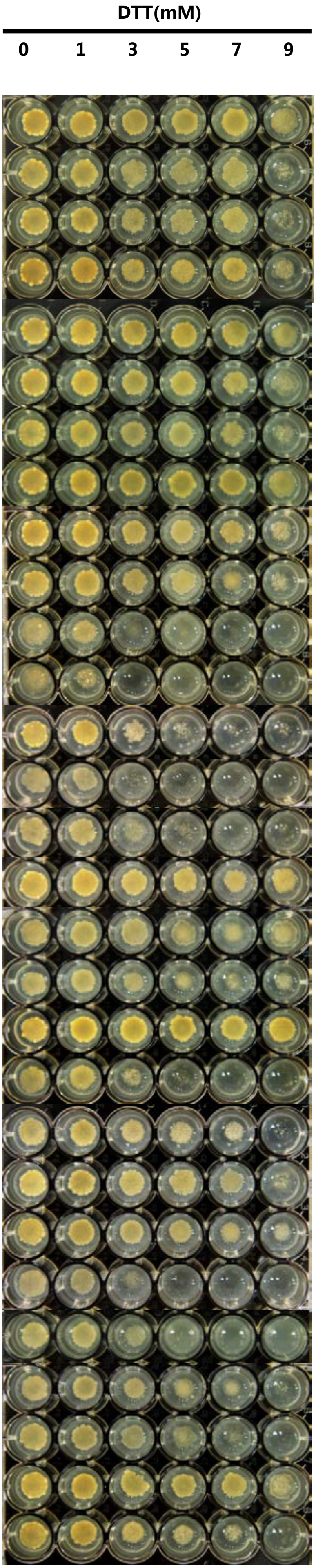

No.	Locus	Gene Product Names	MEDIAN Log2(Fold_Change)	STDEV
021	NCU08635	hypothetical protein	2.69	0.03
022	NCU10763	small oligopeptide transporter	2.63	0.38
023	NCU05535	hypothetical protein	2.6	0.11
024	NCU04168	hypothetical protein	2.58	0.28
025	NCU01231	carboxylic acid transporter	2.56	0.14
026	NCU04167	hypothetical protein	2.56	0.7
027	NCU05303	hypothetical protein	2.54	0.35
028	NCU00449	hypothetical protein	2.53	0.13
029	NCU04169	hypothetical protein	2.53	0.05
030	NCU02175	phosphatidyl inositol-specific phospholipase C	2.51	0.08
031	NCU09760	hypothetical protein	2.45	0.05
032	NCU08634	hypothetical protein	2.43	0.01
033	NCU00782	pantothenate transporter liz1	2.4	0.04
034	NCU02701	dipeptidyl peptidase	2.36	0.37
035	NCU03646	hypothetical protein	2.36	0.33
036	NCU04467	hypothetical protein	2.36	0.06
037	NCU07311	hypothetical protein	2.31	0.35
038	NCU08607	ER-Golgi intermediate compartment protein 3	2.3	0.3
039	NCU10506	triacylglycerol lipase FGL2	2.29	0.64
040	NCU00290	ABC transporter	2.29	0.17
041	NCU07326	hypothetical protein	2.27	0.17
042	NCU03604	hypothetical protein	2.26	0.4
043	NCU09785	hypothetical protein	2.21	0.47
044	NCU09390	tetrahydroxynaphthalene reductase	2.2	0.21
045	NCU03776	ABC multidrug transporter	2.2	0.77
046	NCU09169	NmrA family transcriptional regulator	2.2	0.85
047	NCU01320	glutathione S-transferase	2.19	0.4
048	NCU07817	non-anchored cell wall protein 3	2.19	0.34
049	NCU04170	hypothetical protein	2.19	0.58

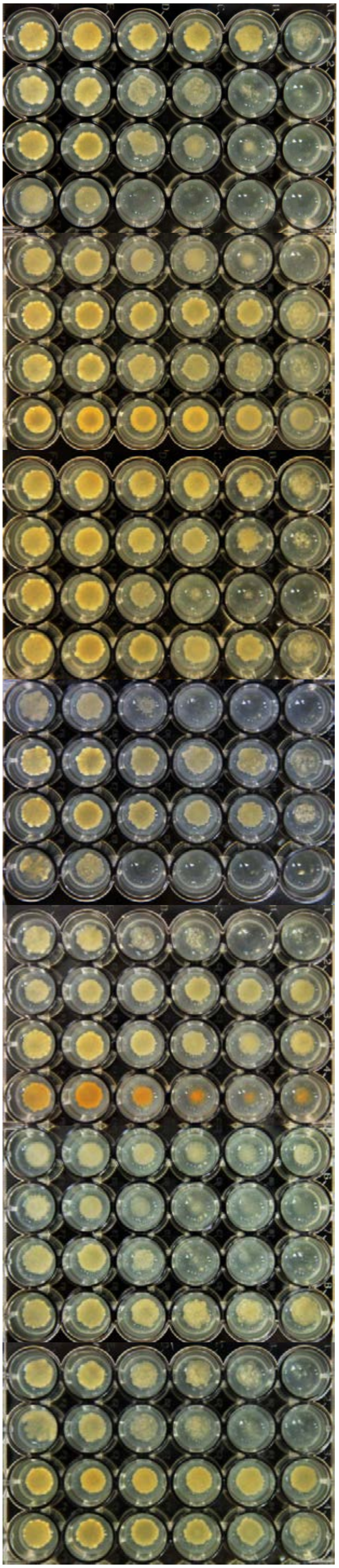

No.	Locus	Gene Product Names	MEDIAN Log2(Fold_Change)	STDEV
050	NCU00055	catechol 1,2-dioxygenase 1	2.19	0.07
051	NCU05822	hypothetical protein	2.17	0.44
052	NCU03248	hypothetical protein	2.16	0.07
053	NCU10809	hypothetical protein	2.14	0.09
054	NCU02932	hypothetical protein	2.12	0.34
055	NCU05236	hypothetical protein	2.1	0.11
056	NCU00129	AMP-binding domain containing protein	2.1	0.2
057	NCU04287	hypothetical protein	2.08	0.5
058	NCU00774	hypothetical protein	2.06	0.41
059	NCU04034	hypothetical protein	2.05	0
060	NCU05498	hypothetical protein	2.03	0.12
061	NCU10055	opsin-1	2.03	0.33
062	NCU08860	hypothetical protein	2.02	0.07
063	NCU02455	FKBP-type peptidyl-prolyl cis-trans isomerase	2.02	0.09
064	NCU00054	hypothetical protein	2.01	0.03
065	NCU03221	PX domain-containing protein	1.99	0.94
066	NCU07359	hypothetical protein	1.99	0.31
067	NCU03518	hypothetical protein	1.99	0.23
068	NCU03318	hypothetical protein	1.96	0.32
069	NCU03967	vivid PAS protein VVD	1.96	0.33
070	NCU00499	all development altered-1	1.95	0.37
071	NCU09786	hypothetical protein	1.92	0.67
072	NCU01815	hypothetical protein	1.92	0.28
073	NCU06860	MFS multidrug transporter	1.91	0.07
074	NCU08487	hypothetical protein	1.9	0.3
075	NCU04045	hypothetical protein	1.88	0.33
076	NCU02913	DNA repair protein rad5	1.86	0.38
077	NCU08491	hypothetical protein	1.85	0.24

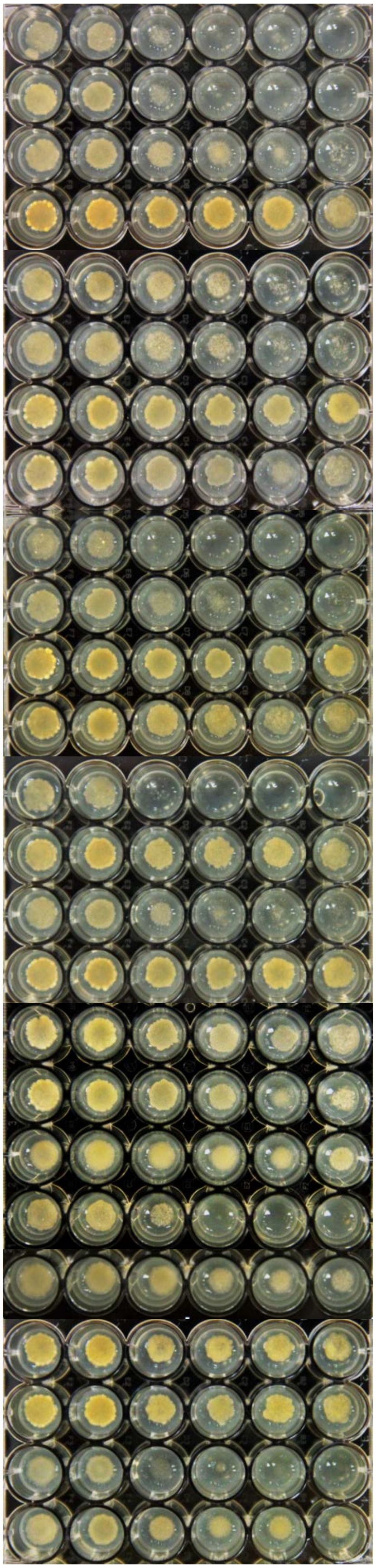

No.	DTT(mM) 0 1 3 5 7 9	Locus	Gene Product Names	MEDIAN Log2(Fold_Change)	STDEV
078		NCU05134	hypothetical protein	1.84	1.04
079		NCU01076	hypothetical protein	1.83	0.2
080		NCU04491	hypothetical protein	1.83	0.11
081		NCU05864	hypothetical protein	1.81	0.08
082		NCU06583	hypothetical protein	1.8	0.14
083		NCU05565	hypothetical protein	1.8	0.03
084		NCU05639	hypothetical protein	1.8	0.5
085		NCU02512	hypothetical protein	1.79	0.62
086		NCU01835	hypothetical protein	1.79	0.57
087		NCU09199	tyrosinase	1.78	0.17
088		NCU04346	hypothetical protein	1.78	0.38
089		NCU02490	DnaJ domain-containing protein	1.77	0.2
090		NCU07894	oligopeptide transporter 2	1.77	0.2
091		NCU07769	hypothetical protein	1.75	0.19
092		NCU08398	aldose 1-epimerase	1.74	0.33
093		NCU08998	4-aminobutyrate aminotransferase	1.73	0.12
094		NCU02153	hypothetical protein	1.73	0.06
095		NCU03287	hypothetical protein	1.71	0.41
096		NCU00870	hypothetical protein	1.68	0.17
097		NCU04726	beta-N-acetylglucosaminidase	1.68	0.09
098		NCU05693	interferon-induced GTP-binding protein Mx2	1.66	0.23
099		NCU04223	hypothetical protein	1.66	0.38
100		NCU09041	L-xylulose reductase	1.65	0.68
101		NCU03686	oxidase assembly protein 2	1.65	0
102		NCU07404	alpha-mannosidase	1.65	0.01

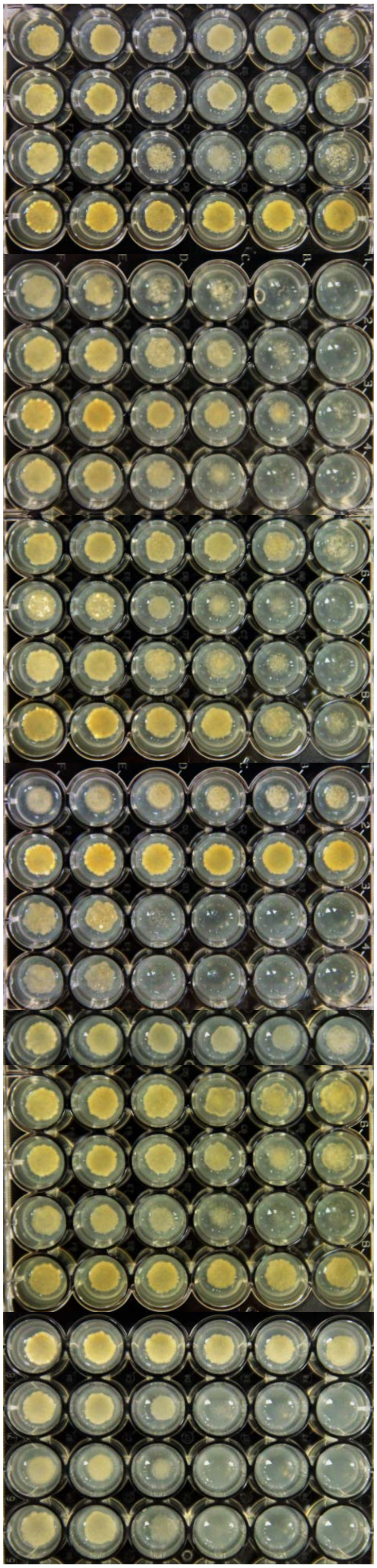

No.	Locus	Gene Product Names	MEDIAN Log2(Fold_Change)	STDEV
103	NCU04272	ZZ type zinc finger domain -containing protein	1.64	0.04
104	NCU06073	hypothetical protein	1.64	0.06
105	NCU04885	alpha-xylosidase	1.64	0.11
106	NCU02547	hypothetical protein	1.63	0.32
107	NCU02134	transcription factor	1.63	0
108	NCU01794	hypothetical protein	1.62	0.28
109	NCU04262	hypothetical protein	1.61	0.28
110	NCU09886	hypothetical protein	1.6	0.59
111	NCU02515	dipeptidyl aminopeptidase	1.6	0.43
112	NCU00289	TAH-1	1.59	0.24
113	NCU02885	hypothetical protein	1.59	0.44
114	NCU04003	ERP1 protein	1.58	0.07
115	NCU03171	small oligopeptide transporter	1.57	0.06
116	NCU00138	pheromone receptor pre-1	1.57	0.08
117	NCU06125	hypothetical protein	1.55	0.02
118	NCU03569	hypothetical protein	1.54	0.39
119	NCU00024	yippee zinc-binding protein	1.54	0.4
120	NCU00821	sugar transporter	1.54	0.13
121	NCU00025	integral membrane protein	1.53	0.12
122	NCU01200	peptidyl-prolyl cis-trans isomerase B	1.52	0.17
123	NCU07121	modin	1.52	0.11
124	NCU00332	hypothetical protein	1.51	0.17
125	NCU05980	carboxypeptidase S1	1.5	0.32
126	NCU05133	udp-glucose 4-epimerase	1.5	0.39
127	NCU01342	ER vesicle protein 25	1.5	0.14

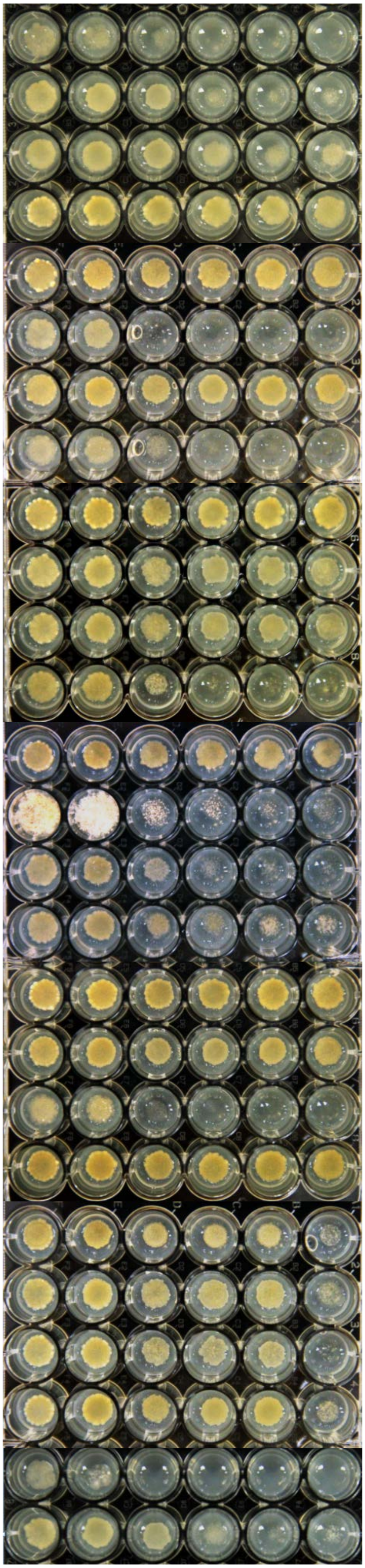

No.	Locus	Gene Product Names	MEDIAN Log2(Fold_Change)	STDEV
128	NCU03465	hypothetical protein	1.49	0.25
129	NCU09698	hypothetical protein	1.48	0.18
130	NCU06342	phospholipase D	1.48	0.18
131	NCU03091	hypothetical protein	1.48	0.2
132	NCU06250	F-box/LRR repeat containing protein 2	1.47	0.17
133	NCU07079	hypothetical protein	1.47	0.15
134	NCU00515	hypothetical protein	1.47	0.14
135	NCU03263	transmembrane protein	1.47	0.19
136	NCU03328	endoglucanase II	1.46	0.44
137	NCU01327	hypothetical protein	1.46	0.04
138	NCU01878	vesicle-mediated transporter	1.45	0.11
139	NCU07473	glycosyltransferase family 28 domain-containing protein	1.45	0.43
140	NCU08127	hypothetical protein	1.45	0.34
141	NCU02582	RCO3	1.45	0.28
142	NCU04808	hypothetical protein	1.44	0.3
143	NCU05395	hypothetical protein	1.43	0.13
144	NCU08705	hypothetical protein	1.43	0.24
145	NCU04550	hypothetical protein	1.43	0.09
146	NCU00373	hypothetical protein	1.42	0.45
147	NCU01426	hypothetical protein	1.41	0.08
148	NCU00046	leucine Rich Repeat domain containing protein	1.4	0.19
149	NCU06200	DUF1682 domain-containing protein	1.4	0.07
150	NCU03243	inositol phosphosphingolipid phospholipase C	1.4	0.15
151	NCU05386	hypothetical protein	1.4	0.06
152	NCU03043	C2H2 finger domain-containing protein FlbC	1.39	0.01
153	NCU08599	hypothetical protein	1.38	0.01

DTT(mM) 0 1 3 5 7 9

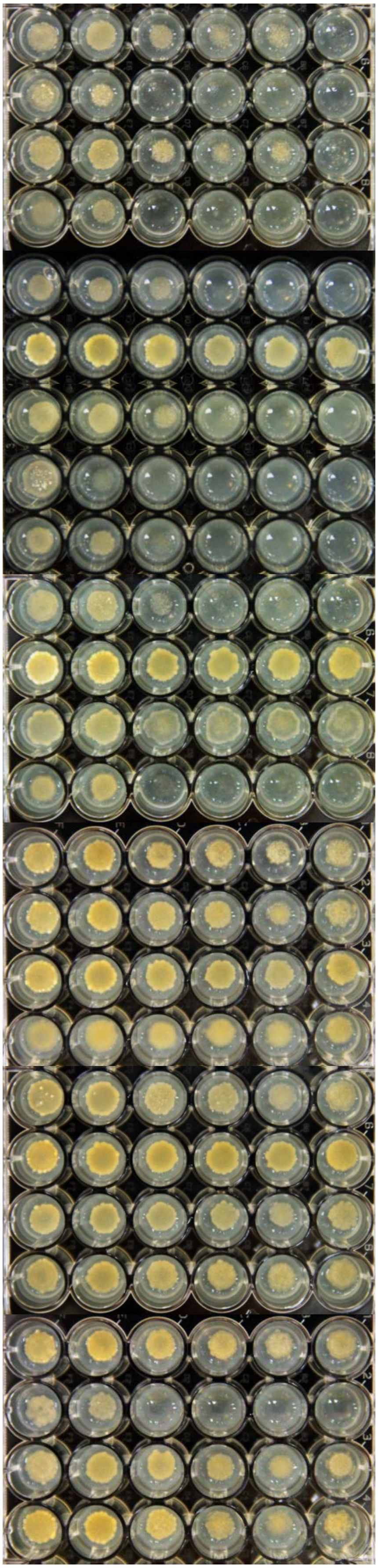

No.	Locus	Gene Product Names	MEDIAN Log2(Fold_Change)	STDEV
154	NCU02945	hypothetical protein	1.36	0.13
155	NCU03654	hypothetical protein	1.34	0.04
156	NCU06380	zinc transporter YKE4	1.34	0.03
157	NCU04736	plasma membrane calcium -transporting ATPase 3	1.34	0.27
158	NCU04119	SNARE complex subunit	1.33	0.05
159	NCU10732	mitochondrial dicarboxylate transporter	1.32	0.3
160	NCU03783	transmembrane domain transporter	1.31	0.03
161	NCU07293	hypothetical protein	1.3	0.02
162	NCU05064	C2H2 transcription factor	1.3	0.12
163	NCU08779	hypothetical protein	1.3	0.01
164	NCU02814	serine/threonine-protein kinase chk2	1.29	0.03
165	NCU07530	transporter smf2	1.29	0.03
166	NCU03699	zinc finger containing protein	1.29	0.03
167	NCU09215	hypothetical protein	1.28	0.02
168	NCU04657	1-aminocyclopropane-1-carboxylate deaminase	1.28	0.07
169	NCU09976	rhamnogalacturonan acetylesterase	1.27	0.05
170	NCU03800	endosomal cargo receptor	1.26	0.08
171	NCU05606	glucosidase 2 subunit beta	1.26	0.11
172	NCU09325	WD domain-containing protein	1.26	0.01
173	NCU00600	small Rho-type GTPase	1.25	0.11
174	NCU08820	hypothetical protein	1.25	0.1
175	NCU06123	phosphoketolase	1.25	0
176	NCU00215	hypothetical protein	1.24	0.09
177	NCU06691	hypothetical protein	1.23	0.03
178	NCU04390	fungal specific transcription factor	1.22	0.11

DTT(mM): 0 1 3 5 7 9

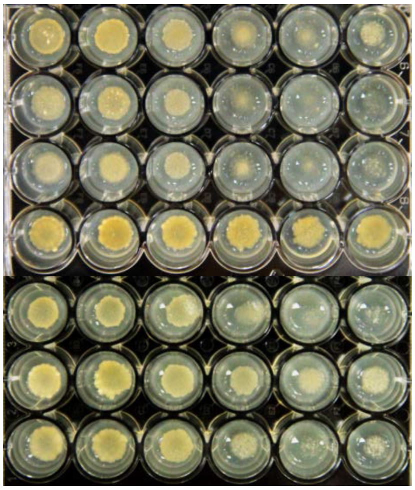

No.	Locus	Gene Product Names	MEDIAN Log2(Fold_Change)	STDEV
179	NCU04203	glucosidase II alpha subunit	1.21	0.18
180	NCU09160	HPP family protein	1.20	0.12
181	NCU01924	hypothetical protein	1.19	0.04
182	NCU08371	hypothetical protein	1.18	0.06
183	NCU02576	C6 finger domain-containing protein	1.17	0.01
184	NCU01039	hypothetical protein	1.16	0.02
185	NCU04905	hypothetical protein	1.15	0.01

ER Stress Core Targets

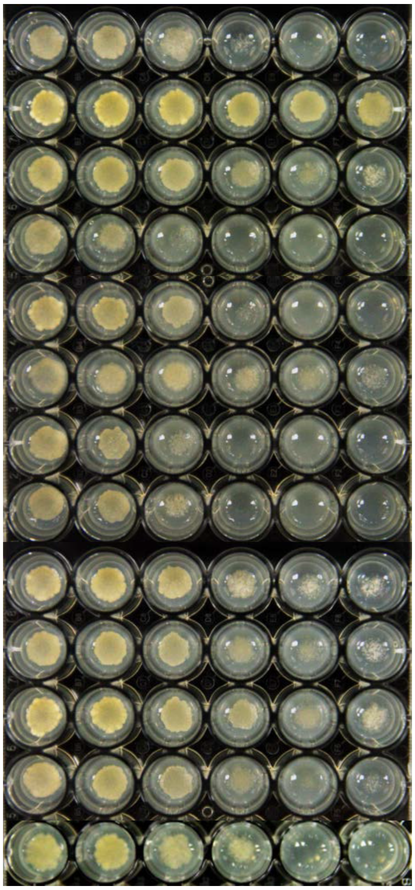

No.	Locus	Gene Product Names	MEDIAN Log2(Fold_Change)	STDEV
186	NCU09402	hypothetical protein	5.21	0.65
187	NCU03636	hypothetical protein	4.8	0.75
188	NCU04046	hypothetical protein	4.56	1.89
189	NCU05277	hypothetical protein	4.54	2.7
190	NCU00968	SIL1	4.11	0.77
191	NCU10721	solute carrier family 35 member B1 protein	3.97	0.19
192	NCU09485	chaperone dnaK	3.8	0.4
193	NCU10039	hypothetical protein	3.79	0.22
194	NCU06237	hypothetical protein	3.76	0.66
195	NCU00965	hypothetical protein	3.67	0.42
196	NCU04194	hypothetical protein	3.46	0.24
197	NCU11102	SCJ1	3.4	0.15
198	NCU07037	hypothetical protein	3.34	1.68

DTT(mM)

0 1 3 5 7 9

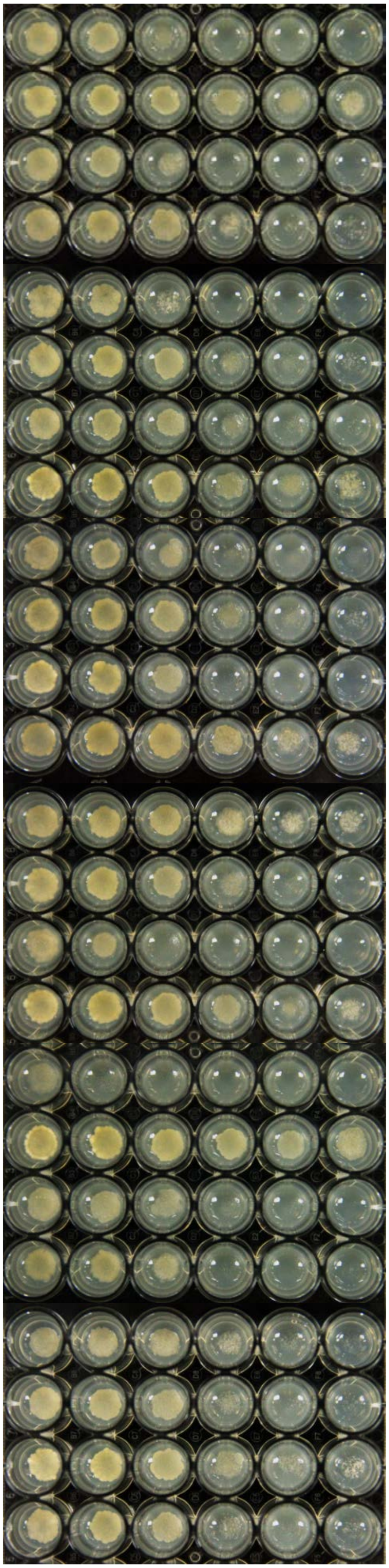

No.	Locus	Gene Product Names	MEDIAN Log2(Fold_Change)	STDEV
199	NCU05780	glutathione transferase	3.33	0.9
200	NCU02653	allantoate permease	3.33	0.99
201	NCU08764	hypothetical protein	3.19	0.36
202	NCU01268	ubiquitin-conjugating enzyme E2 6	3.14	0.31
203	NCU04360	hypothetical protein	3.07	1.29
204	NCU00146	ER-associated proteolytic system protein Der1	3.05	0.3
205	NCU06400	amidohydrolase	3.02	1.8
206	NCU07879	mitochondrial metal t ransporter 2	3	1.53
207	NCU08986	hypothetical protein	2.97	1.53
208	NCU02681	translocation protein	2.93	0.17
209	NCU00813	disulfide isomerase	2.91	0.34
210	NCU03459	UBA domain-containing protein Ucp14	2.89	0.34
211	NCU03152	DUF1348 domain-containing protein	2.87	0.71
212	NCU02663	L-lysine 2,3-aminomutase	2.86	0.49
213	NCU02877	hypothetical protein	2.82	1.26
214	NCU02550	alpha-galactosidase	2.82	0.12
215	NCU10762	UDP-N-acetyl-glucosamine-1-P transferase Alg7	2.8	0.88
216	NCU00282	hypothetical protein	2.77	0.89
217	NCU02424	DnaJ and TPR domain-containing protein	2.76	0.31
218	NCU04847	cyclin	2.72	0.89
219	NCU03305	ca-transporting ATPase sarcoplasmic/ER type	2.71	0.18
220	NCU05315	hypothetical protein	2.69	0.32
221	NCU07545	DUF1183 domain-containing protein	2.67	0.79
222	NCU09283	acetyltransferase	2.66	0.6

DTT(mM)

0 1 3 5 7 9

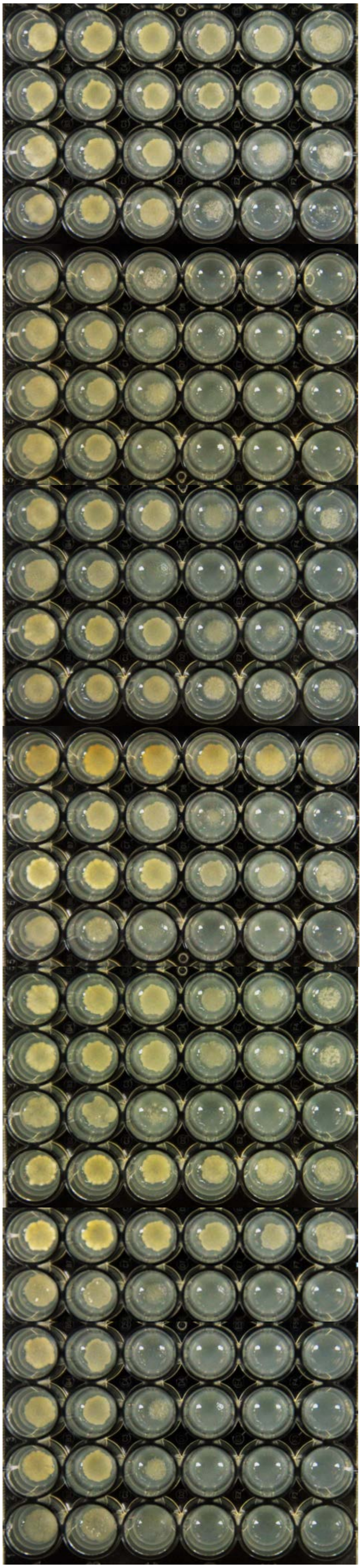

No.	Locus	Gene Product Names	MEDIAN Log2(Fold_Change)	STDEV
223	NCU03637	CaaX prenyl protease Ste24	2.66	0.17
224	NCU01193	hypothetical protein	2.64	0.75
225	NCU01898	hypothetical protein	2.64	0.48
226	NCU08379	hypothetical protein	2.63	0.24
227	NCU08641	hypothetical protein	2.62	1.19
228	NCU06386	dolichyl-phosphate beta-glucosyltransferase	2.61	0.53
229	NCU06763	hypothetical protein	2.58	0.45
230	NCU06230	serine/threonine protein kinase	2.57	0.71
231	NCU04633	RING finger protein	2.56	0.26
232	NCU08229	hypothetical protein	2.56	1.02
233	NCU00162	hypothetical protein	2.51	0.46
234	NCU08847	hypothetical protein	2.5	0.72
235	NCU01928	UBX domain-containing protein	2.46	0.16
236	NCU02750	PH domain-containing protein	2.46	1.03
237	NCU02909	chitosanase	2.45	0.56
238	NCU03083	hypothetical protein	2.42	0.25
239	NCU00598	thioredoxin	2.41	0.26
240	NCU02391	protein transporter sec-24	2.4	0.42
241	NCU03819	COPII coat assembly protein sec-16	2.4	0.1
242	NCU09321	sucrose transporter	2.39	0.57
243	NCU01275	oxidoreductase	2.38	0.41
244	NCU02636	ubiquitin conjugating enzyme	2.32	0.28
245	NCU09101	hypothetical protein	2.29	0.27
246	NCU06708	protein transporter sec22	2.27	0.45
247	NCU04544	hypothetical protein	2.27	0.61
248	NCU04316	pheromone processing carboxypeptidase Kex1	2.25	0.29

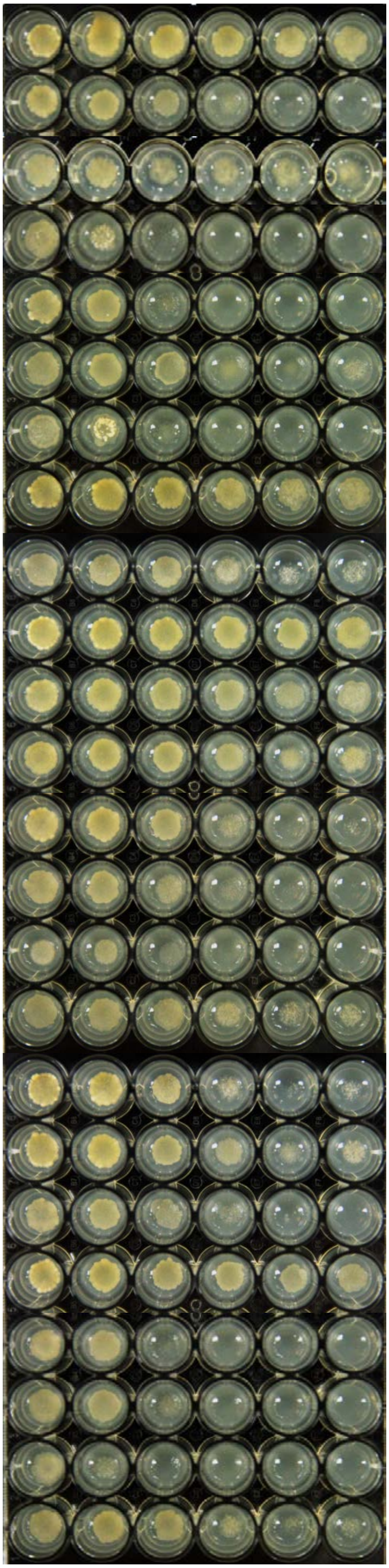

No.	Locus	Gene Product Names	MEDIAN Log2(Fold_Change)	STDEV
249	NCU03506	hypothetical protein	2.21	0.56
250	NCU00214	hypothetical protein	2.2	0.36
251	NCU08091	hypothetical protein	2.15	1.12
252	NCU01296	hypothetical protein	2.14	0.17
253	NCU09333	zinc finger transcription factor ace1	2.14	0.38
254	NCU03694	hypothetical protein	2.11	0.25
255	NCU04990	protein serine/threonine kinase	2.09	0.45
256	NCU01146	signal sequence receptor alpha chain	2.08	0.17
257	NCU03681	hypothetical protein	2.05	0.33
258	NCU04193	hypothetical protein	2.03	1.42
259	NCU06704	hypothetical protein	2.02	0.59
260	NCU10852	exochitinase	1.98	0.55
261	NCU03079	hypothetical protein	1.98	0.52
262	NCU03308	hypothetical protein	1.98	0.41
263	NCU02491	DNAJ domain-containing protein	1.97	0.43
264	NCU10035	hypothetical protein	1.96	0.39
265	NCU03897	RNA binding effector protein Scp160	1.94	0.32
266	NCU03317	glucosyltransferase	1.94	0.34
267	NCU04898	cation-transporting ATPase 4	1.92	0.29
268	NCU04293	COPII-coated vesicle protein	1.92	0.25
269	NCU02138	hypothetical protein	1.91	0.34
270	NCU00585	phytoene synthase	1.91	0.83
271	NCU04192	vacuolar aspartyl aminopeptidase Lap4	1.9	0.14
272	NCU02718	hypothetical protein	1.9	0.65

DTT(mM)

0 1 3 5 7 9

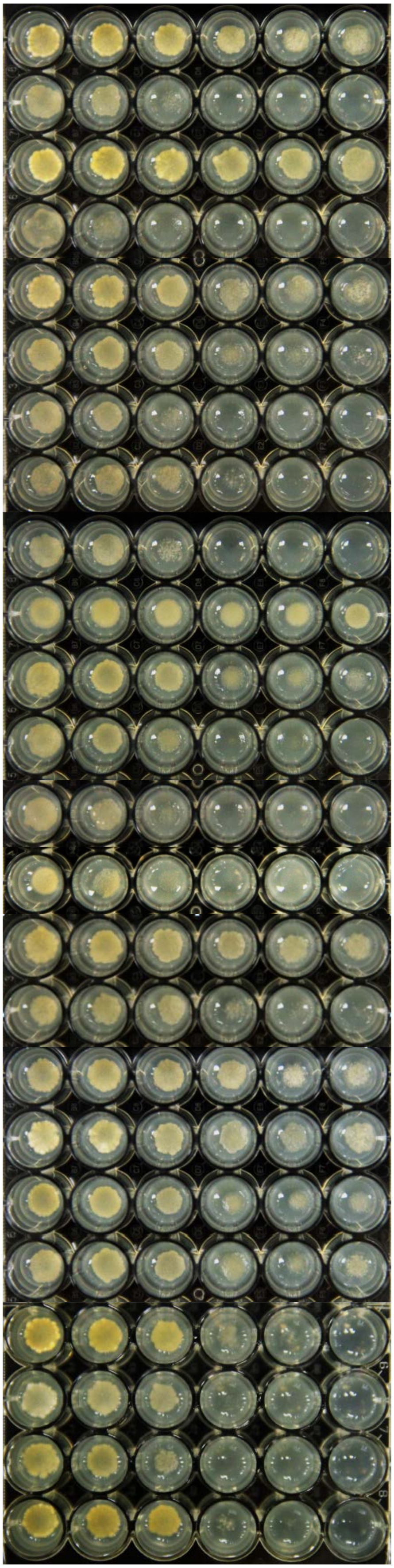

No.	Locus	Gene Product Names	MEDIAN Log2(Fold_Change)	STDEV
273	NCU04255	hypothetical protein	1.9	0.42
274	NCU09241	hypothetical protein	1.89	0.76
275	NCU10789	ubiquitin-protein ligase Sel1/Ubx2	1.88	0.3
276	NCU07125	cytomegalovirus gH-receptor family protein	1.85	0.4
277	NCU02463	bax Inhibitor family protein	1.84	0.56
278	NCU09882	metacaspase-1A	1.81	0.28
279	NCU06166	alpha-1,2-mannosyltransferase	1.81	0.56
280	NCU08743	inorganic phosphate transporter	1.8	0.45
281	NCU00182	integral membrane protein	1.76	0.32
282	NCU09263	hypothetical protein	1.76	0.37
283	NCU02164	hypothetical protein	1.76	1.52
284	NCU09403	NmrA family protein	1.74	0.34
285	NCU04554	endochitinase 1	1.71	0.45
286	NCU08807	DNA-binding protein cre-1	1.69	1.04
287	NCU00188	ATG1 protein	1.69	0.1
288	NCU00814	hypothetical protein	1.68	0.45
289	NCU00026	hypothetical protein	1.65	0.33
290	NCU04610	oxysterol binding protein	1.65	0.46
291	NCU00490	DUF803 domain membrane protein	1.65	0.42
292	NCU08957	hypothetical protein	1.62	0.5
293	NCU06460	acid phosphatase	1.62	1.15
294	NCU09108	hypothetical protein	1.61	0.9
295	NCU04457	DUF803 domain membrane protein	1.6	0.11
296	NCU02307	hypothetical protein	1.6	0.43

DTT(mM)

0 1 3 5 7 9

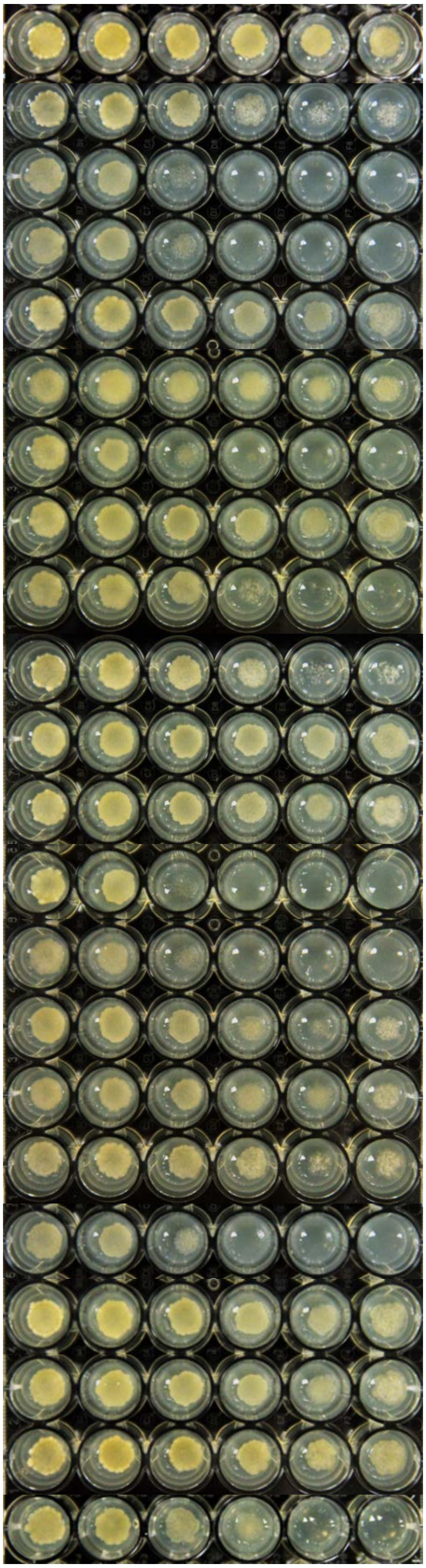

No.	Locus	Gene Product Names	MEDIAN Log2(Fold_Change)	STDEV
297	NCU00916	membrane bound cation transporter	1.61	0.31
298	NCU00481	a-pheromone processing metallopeptidase Ste23	1.59	0.35
299	NCU05276	Yip1 domain family	1.58	0.49
300	NCU01879	hypothetical protein	1.58	0.26
301	NCU01947	hypothetical protein	1.56	0.17
302	NCU06710	ClpTM1 domain-containing protein	1.54	0.22
303	NCU00918	hypothetical protein	1.53	0.09
304	NCU09806	hypothetical protein	1.51	0.24
305	NCU07425	hypothetical protein	1.51	0.34
306	NCU07273	hypothetical protein	1.49	0.55
307	NCU03881	hypothetical protein	1.48	0.27
308	NCU06242	lectin family integral membrane protein	1.47	0.19
309	NCU01886	hypothetical protein	1.45	0.44
310	NCU00898	hypothetical protein	1.44	0.07
311	NCU03064	RSC complex subunit Rsc7	1.44	0.15
312	NCU00725	epsin-3	1.43	0.14
313	NCU05990	cell surface receptor/ MFS transporter	1.42	0.28
314	NCU03710	integral membrane protein	1.35	1.02
315	NCU00035	diacylglycerol O-acyltransferase	1.32	0.07
316	NCU02422	ATG9 protein	1.31	0.21
317	NCU09252	hypothetical protein	1.3	0.2
318	NCU09711	hypothetical protein	1.3	0.7

Acute Stress Specific Targets

DTT(mM): 0 1 3 5 7 9

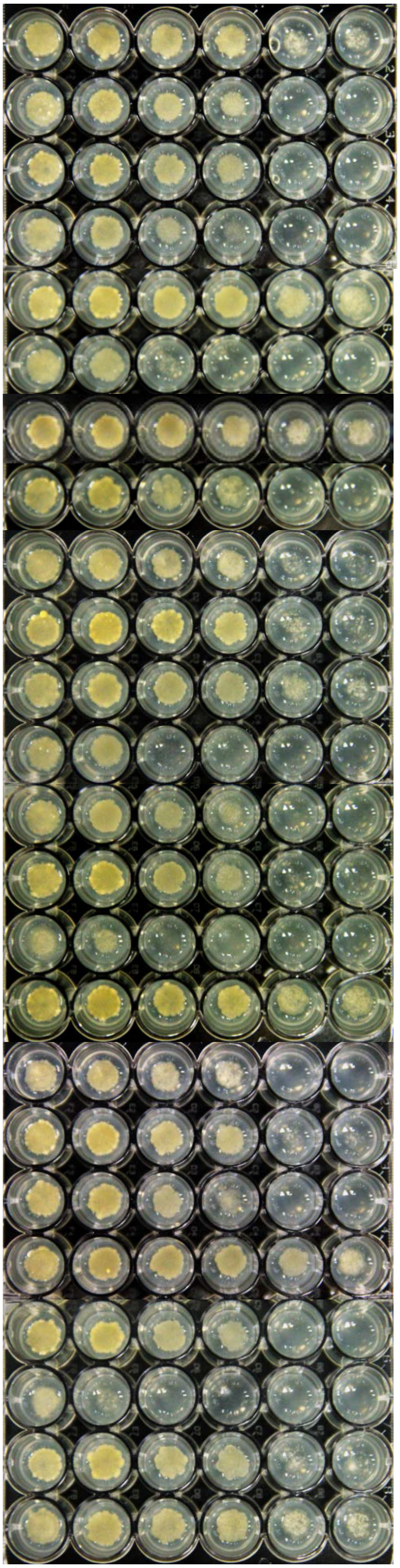

No.	Locus	Gene Product Names	MEDIAN Log2(Fold_Change)	STDEV
319	NCU11098	UPF0052 domain-containing protein	5.71	1.59
320	NCU04641	FAD dependent oxidoreductase	5.67	1.3
321	NCU04619	hypothetical protein	5.38	1.07
322	NCU00868	hypothetical protein	4.78	0.29
323	NCU00017	hypothetical protein	4.71	0.58
324	NCU01070	hypothetical protein	4.68	0.27
325	NCU10239	hypothetical protein	4.66	3.22
326	NCU02700	hypothetical protein	4.32	0.11
327	NCU00869	hypothetical protein	4.28	0.13
328	NCU03213	mannosylphosphorylation protein	4.27	2.17
329	NCU00473	hypothetical protein	4.26	2.2
330	NCU03921	mitochondrial chaperone bcs1	4.25	0.24
331	NCU04218	RTA1 domain-containing protein	4.03	1.91
332	NCU07840	hypothetical protein	4.02	0.3
333	NCU04058	hypothetical protein	4	0.36
334	NCU04016	phosphoglycerate mutase	4	2.3
335	NCU08390	hypothetical protein	3.89	2.92
336	NCU00247	hypothetical protein	3.84	0.13
337	NCU07134	hypothetical protein	3.77	1.09
338	NCU04639	non-classical export protein Nce102	3.72	1.56
339	NCU07030	hypothetical protein	3.71	0.47
340	NCU04583	acetyltransferase	3.67	3.19
341	NCU00855	hypothetical protein	3.64	0.64
342	NCU00865	oxalate decarboxylase oxdC	3.6	1.85

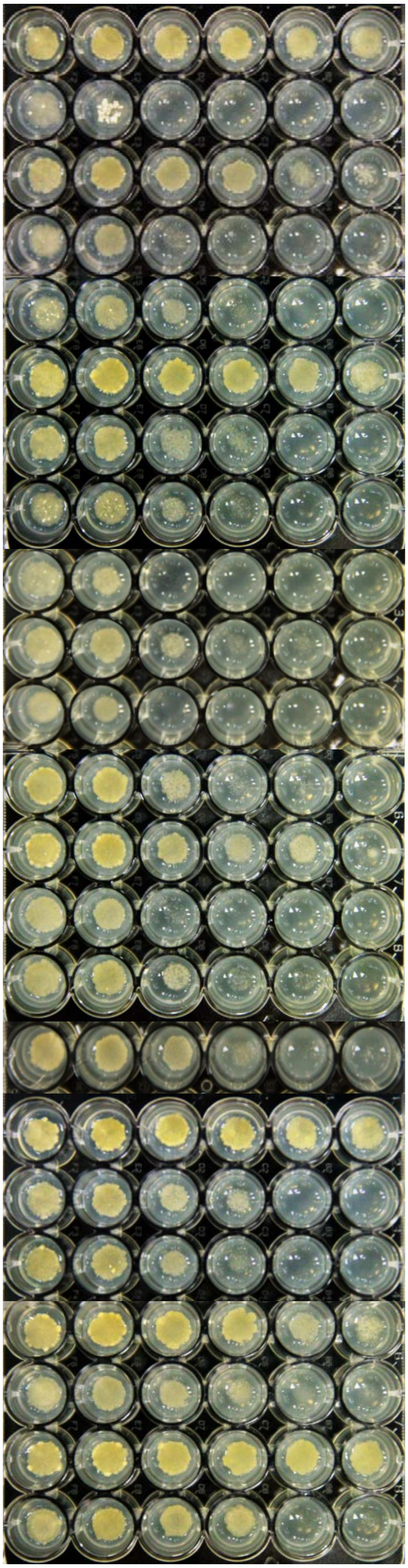

No.	Locus	Gene Product Names	MEDIAN Log2(Fold_Change)	STDEV
343	NCU01147	tyrosine decarboxylase	3.58	0.47
344	NCU07135	hypothetical protein	3.57	1.07
345	NCU04421	annexin XIV	3.49	1.14
346	NCU03205	hypothetical protein	3.48	1.22
347	NCU11050	DUF455 domain -containing protein	3.42	1.7
348	NCU06884	hypothetical protein	3.39	0.11
349	NCU04256	hypothetical protein	3.34	1.04
350	NCU06425	hypothetical protein	3.26	0.6
351	NCU08674	Pentatricopeptide repeat protein	3.13	1.15
352	NCU01862	SWIRM domain-containing protein FUN19	3.13	0.02
353	NCU01069	amphiphysin-like lipid raft protein	3.11	0.53
354	NCU08271	GTP-binding protein yptV5	3.07	0.09
355	NCU01081	hypothetical protein	3.07	1.39
356	NCU06264	mutagen sensitive-53	3.03	0.75
357	NCU00248	hypothetical protein	3.03	1.1
358	NCU05922	hypothetical protein	3.03	0.54
359	NCU04760	golgi apparatus membrane protein tvp-18	2.99	1.24
360	NCU08720	hypothetical protein	2.98	0.3
361	NCU05264	pyrimidine 5'-nucleotidase	2.98	0.97
362	NCU04112	hypothetical protein	2.93	0.3
363	NCU04260	oxidoreductase domain-containing protein	2.91	1.33
364	NCU03368	hypothetical protein	2.89	2.33
365	NCU03684	hypothetical protein	2.88	0.2

DTT(mM)

0 1 3 5 7 9

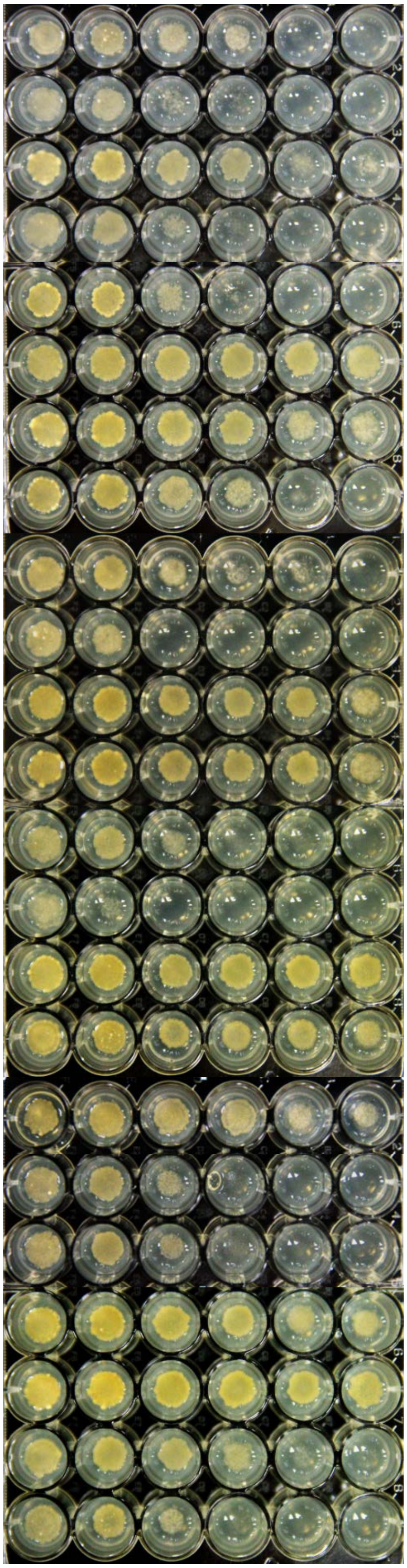

No.	Locus	Gene Product Names	MEDIAN Log2(Fold_Change)	STDEV
366	NCU00454	hypothetical protein	2.88	0.4
367	NCU08184	hypothetical protein	2.84	1.13
368	NCU04014	hypothetical protein	2.82	1.18
369	NCU02883	hypothetical protein	2.81	1.42
370	NCU01060	hypothetical protein	2.81	0.85
371	NCU00955	monooxygenase	2.8	1.36
372	NCU09282	hypothetical protein	2.78	0.55
373	NCU06862	hypothetical protein	2.75	1.01
374	NCU01629	hypothetical protein	2.74	0.62
375	NCU09629	hypothetical protein	2.73	0.42
376	NCU06586	AN1 zinc finger protein	2.72	0.35
377	NCU05534	hypothetical protein	2.71	0.26
378	NCU02390	hypothetical protein	2.69	0.54
379	NCU09513	GTP-binding protein 1	2.67	0.98
380	NCU09674	O-methyltransferase family 3	2.67	0.27
381	NCU01865	hypothetical protein	2.66	0.18
382	NCU01080	glucanase B	2.63	0.15
383	NCU03336	hypothetical protein	2.62	0.43
384	NCU07441	hypothetical protein	2.62	1.15
385	NCU09473	3-ketoacyl-acyl carrier protein reductase	2.6	0.97
386	NCU06265	hypothetical protein	2.6	0.22
387	NCU06926	hypothetical protein	2.58	0.93
388	NCU04161	multidrug resistance-associated protein 5	2.57	0.46

DTT(mM)

0 1 3 5 7 9

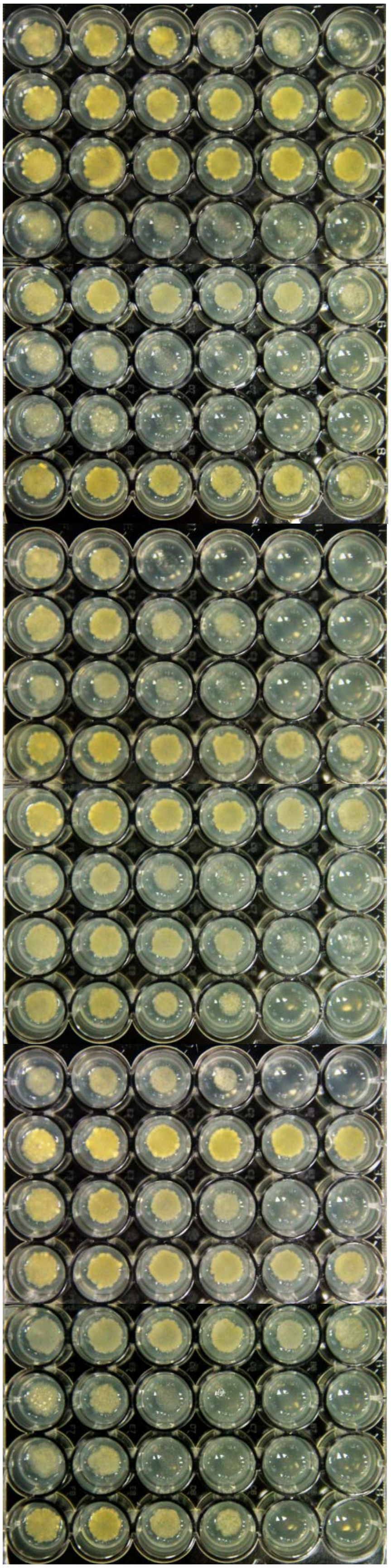

No.	Locus	Gene Product Names	MEDIAN Log2(Fold_Change)	STDEV
389	NCU00322	hypothetical protein	2.56	1.45
390	NCU07888	hypothetical protein	2.55	0.13
391	NCU06944	hypothetical protein	2.55	0.2
392	NCU03355	calpain-5	2.53	0.33
393	NCU09281	alpha-glucosidase	2.52	0.02
394	NCU02142	hypothetical protein	2.5	0.35
395	NCU04924	HAD-superfamily hydrolase	2.5	0.23
396	NCU10400	phospholipase PldA	2.49	1.53
397	NCU07741	1-acyl-sn-glycerol-3-phosphate acyltransferase 2	2.47	0.62
498	NCU01997	ABC transporter	2.46	0.07
499	NCU09986	hypothetical protein	2.42	0.44
400	NCU06095	CP2 transcription factor	2.41	0.29
401	NCU03993	hypothetical protein	2.41	1.63
402	NCU04637	BAR adaptor protein RVS167	2.4	1.13
403	NCU07439	hypothetical protein	2.39	1.23
404	NCU01422	multiprotein-bridging factor 1	2.37	0.18
405	NCU06709	hypothetical protein	2.35	0.89
406	NCU01188	ubiquitin conjugating enzyme	2.35	1.06
407	NCU05309	Cut9 interacting protein Scn1	2.33	1.08
408	NCU03507	hypothetical protein	2.33	0.05
409	NCU06969	phosphoinositide 3-phosphate phosphatase	2.31	1.11
410	NCU00088	4-nitrophenylphosphatase	2.3	1.01
411	NCU03451	hypothetical protein	2.27	0.66
412	NCU01330	SacI domain-containing protein	2.26	0.8

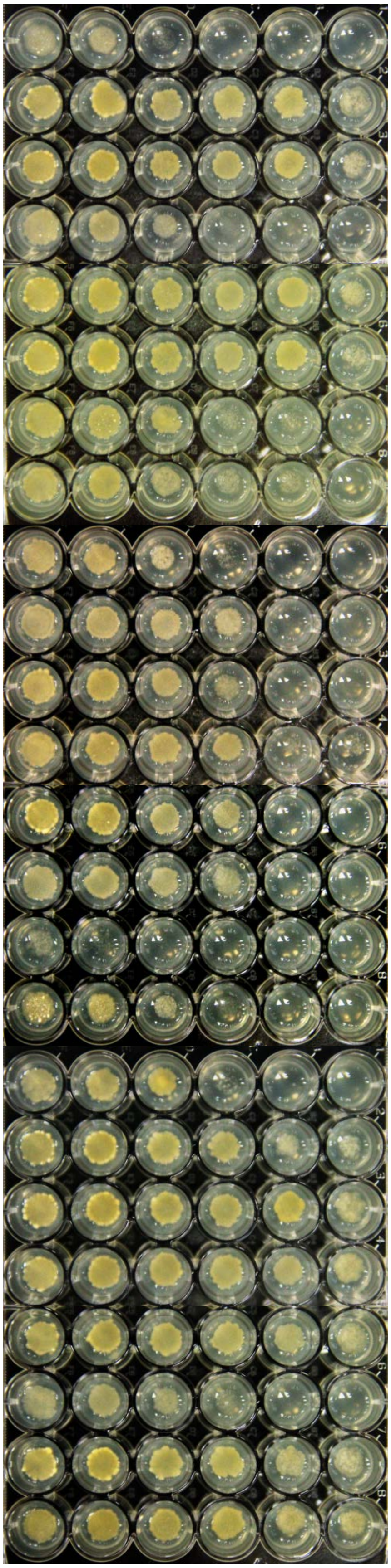

No.	Locus	Gene Product Names	MEDIAN Log2(Fold_Change)	STDEV
413	NCU03773	hypothetical protein	2.26	0.84
414	NCU06034	hypothetical protein	2.26	1.46
415	NCU01818	hypothetical protein	2.26	0.3
416	NCU08938	DNA-3-methyladenine glycosylase	2.25	0.23
417	NCU00121	CLC channel protein	2.25	0.53
418	NCU00604	hypothetical protein	2.24	0.06
419	NCU05873	Amy1	2.23	0.82
420	NCU02544	ABC transporter	2.23	0.32
421	NCU06317	stress response RCI peptide	2.2	1.22
422	NCU03714	thioredoxin	2.2	0.38
423	NCU06401	hypothetical protein	2.19	1.16
424	NCU00399	cell wall protein PhiA	2.19	0.13
425	NCU01064	hypothetical protein	2.19	0.1
426	NCU06616	S-adenosylmethionine-dependent methyltransferase	2.18	0.48
427	NCU06660	plasma membrane proteolipid 3	2.17	0.28
428	NCU04452	12-oxophytodienoate reductase 1	2.13	1.25
429	NCU09692	phosphatidic acid phosphatase beta	2.13	0.65
430	NCU02724	HLH transcription factor	2.13	0.68
431	NCU03647	hypothetical protein	2.12	1.12
432	NCU03698	hypothetical protein	2.12	0.32
433	NCU00684	endonuclease/Exonuclease/ phosphatase	2.12	0.09
434	NCU02386	hypothetical protein	2.11	0.34
435	NCU07434	short-chain dehydrogenase/ reductase SDR	2.08	0
436	NCU06651	AN1-type zinc finger protein	2.08	0.27

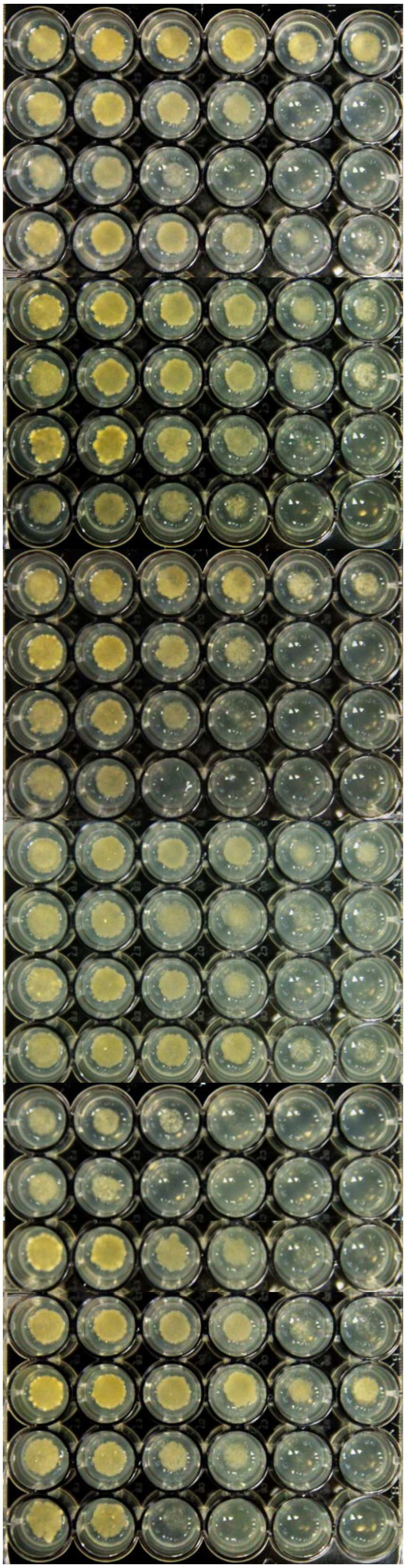

No.	Locus	Gene Product Names	MEDIAN Log2(Fold_Change)	STDEV
437	NCU02703	hypothetical protein	2.07	0.52
438	NCU01833	two-component histidine kinase CHK-1	2.07	0.37
439	NCU00619	hypothetical protein	2.06	0.36
440	NCU08346	serine/threonine protein kinase	2.05	1
441	NCU01927	hypothetical protein	2.04	0.76
442	NCU01883	SH3 domain signaling protein	2.04	0.35
443	NCU02122	HCO3	2.04	0.02
444	NCU08334	hypothetical protein	2.02	0.27
445	NCU05525	DUF833 domain-containing protein	2.02	0.6
446	NCU05010	hypothetical protein	2	0.67
447	NCU06114	hypothetical protein	2	0.06
448	NCU03649	hypothetical protein	2	0.05
449	NCU08391	hypothetical protein	1.99	0.49
450	NCU07063	hypothetical protein	1.96	1.14
451	NCU07966	calcium-transporting ATPase 3	1.96	0.02
452	NCU04605	hypothetical protein	1.96	1.16
453	NCU05514	golgi membrane protein	1.95	0.13
454	NCU04050	cross-pathway control protein 1	1.94	0.34
455	NCU09564	phosphate-repressible phosphate permease	1.93	0.92
456	NCU00631	RING-13 protein	1.93	0.07
457	NCU08621	hypothetical protein	1.91	0.01
458	NCU03989	ADP,ATP carrier protein	1.88	0.74
459	NCU05899	flotillin domain-containing protein	1.88	0.51

DTT(mM)

0 1 3 5 7 9

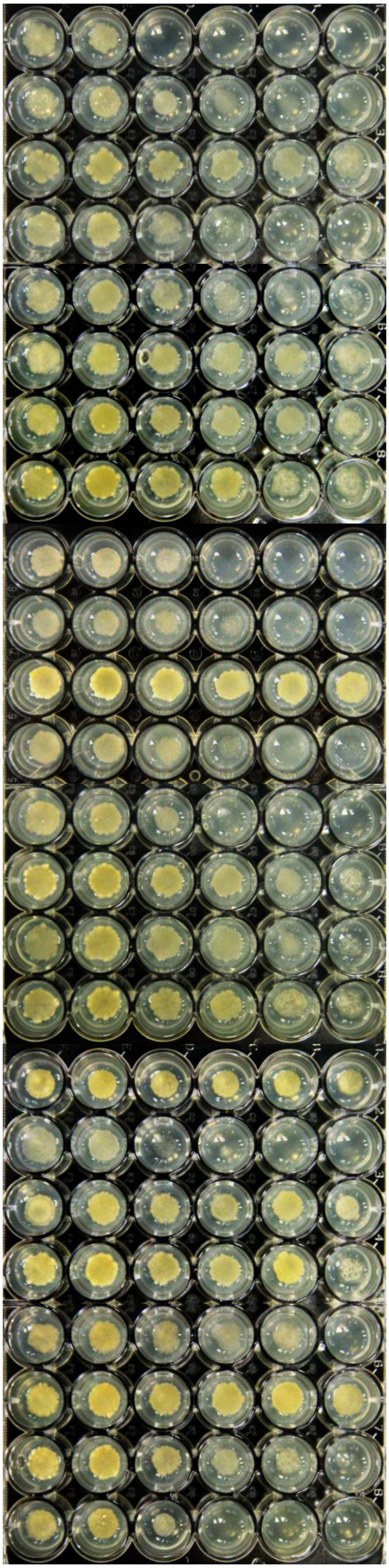

	Locus	Gene Product Names	MEDIAN Log2(Fold_Change)	STDEV
460	NCU05958	hypothetical protein	1.88	0.08
461	NCU01640	C2H2 transcription factor	1.87	0.74
462	NCU05454	glycerol-3-phosphate dehydrogenase	1.85	0.11
463	NCU03571	serine/threonine protein kinase	1.83	0.99
464	NCU07029	hypothetical protein	1.82	0.65
465	NCU01955	autophagocytosis protein Aut1	1.82	0.73
466	NCU09253	DUF907 domain-containing protein	1.82	0.32
467	NCU02120	hypothetical protein	1.81	0.34
468	NCU03650	DNA repair protein RAD16	1.79	0.15
469	NCU03697	FAD binding domain-containing protein	1.79	0.11
470	NCU03312	CorA family metal ion transporter	1.78	0.42
471	NCU07967	hypothetical protein	1.73	0.42
472	NCU05148	hypothetical protein	1.73	0.79
473	NCU07498	DNA excision repair protein Rad2	1.72	0.67
474	NCU10036	golgi matrix protein	1.71	0.5
475	NCU09322	chitin synthase activator	1.71	0.73
476	NCU00579	oxysterol binding protein 1	1.7	0.39
477	NCU09317	hypothetical protein	1.7	0.44
478	NCU03914	glucan 1,3-beta-glucosidase	1.7	0.39
479	NCU04419	2-oxoglutarate-dependent ethylene	1.69	0.12
480	NCU06331	hypothetical protein	1.68	0.31
481	NCU06437	hypothetical protein	1.68	0.32
482	NCU09271	hypothetical protein	1.68	0.14
483	NCU09502	hypothetical protein	1.67	0.01

DTT(mM): 0 1 3 5 7 9

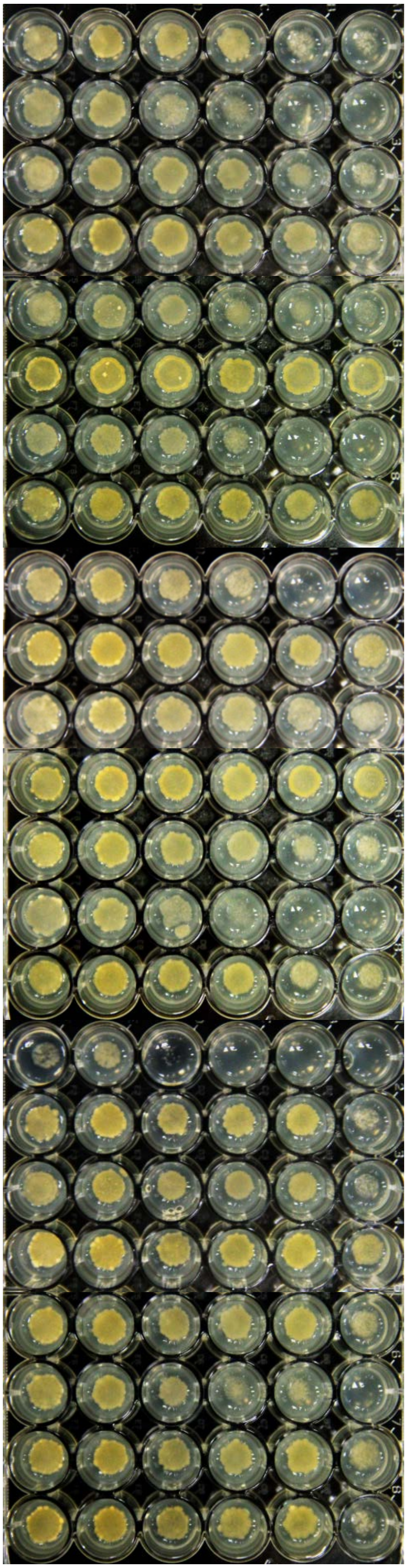

No.	Locus	Gene Product Names	MEDIAN Log2(Fold_Change)	STDEV
484	NCU02834	hypothetical protein	1.67	0.09
485	NCU02203	C2H2 finger domain-containing protein	1.67	0.43
486	NCU01803	arginine-tRNA-protein transferase 1	1.66	0.15
487	NCU03944	WD repeat containing protein 2	1.64	0.4
488	NCU00568	hypothetical protein	1.64	0.4
489	NCU03770	cell wall glycosyl hydrolase Dfg5	1.62	0.1
490	NCU05777	ubiquitin carboxyl-terminal hydrolase 14	1.61	0.54
491	NCU04101	WD domain-containing protein	1.61	0.1
492	NCU03888	DUF500 and SH3 domain-containing protein	1.61	0.67
493	NCU00068	hypothetical protein	1.6	0.18
494	NCU03929	acyl-CoA synthetase	1.55	0.37
495	NCU00388	hypothetical protein	1.55	0.13
496	NCU02747	DUF726 domain-containing protein	1.55	0.2
497	NCU01793	RNA binding domain-containing protein	1.55	0.24
498	NCU00403	dTDP-D-glucose 4,6-dehydratase	1.53	0.1
499	NCU09573	hypothetical protein	1.51	0.47
500	NCU06202	serine/threonine protein kinase	1.51	0.04
501	NCU06273	hypothetical protein	1.5	0.07
502	NCU01646	hypothetical protein	1.5	0.19
503	NCU07495	sphingolipid long chain base-responsive protein	1.5	0.36
504	NCU09159	hypothetical protein	1.47	0.01
505	NCU09748	transcription initiation factor IIA gamma chain	1.47	0.34
506	NCU03591	ATP-dependent bile acid permease	1.46	0.15

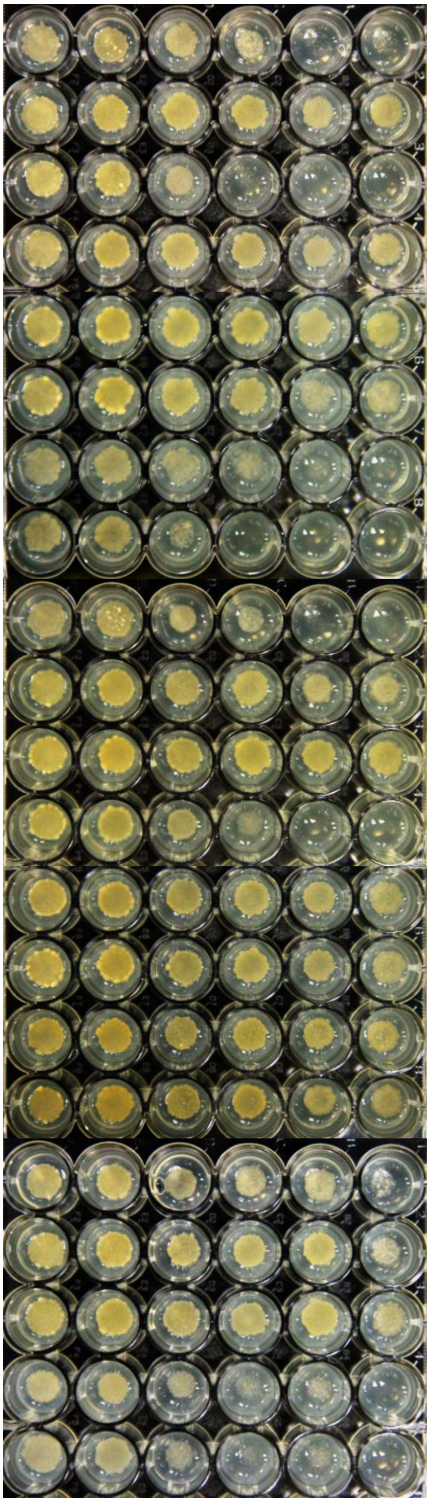

No.	Locus	Gene Product Names	MEDIAN Log2(Fold_Change)	STDEV
507	NCU03381	phosphatidate cytidylyltransferase	1.45	0.31
508	NCU07379	bZIP-type transcription factor	1.45	0.21
509	NCU08273	plasma membrane channel protein	1.44	0.1
510	NCU06698	glycogenin	1.44	0.1
511	NCU06209	hypothetical protein	1.43	0.37
512	NCU04386	hypothetical protein	1.43	0.14
513	NCU01501	mitochondrial peptidyl-tRNA hydrolase Pth2	1.43	0.19
514	NCU04936	UDP-glucose 6-dehydrogenase	1.41	0.08
515	NCU04542	hypothetical protein	1.4	0.28
516	NCU03905	hypothetical protein	1.36	0.24
517	NCU09329	hypothetical protein	1.36	0.08
518	NCU04737	chromatin regulatory protein sir2	1.36	0.03
519	NCU05638	hypothetical protein	1.34	0.17
520	NCU00180	hypothetical protein	1.31	0.03
521	NCU02413	transcription factor prr1	1.31	0.16
522	NCU01181	acyl-CoA dehydrogenase family member 11	1.28	0.2
523	NCU03792	inositol polyphosphate phosphatase	1.24	0.05
524	NCU00351	cript family protein	1.23	0.03
525	NCU02837	pre-mRNA branch site protein p14	1.22	0.02
526	NCU05313	mitochondria fission 1 protein	1.22	0.1
527	NCU07739	YTP1	1.17	0.05
