# Supplementary material for: Genome-wide analysis of the endoplasmic reticulum stress response during lignocellulase production in Neurospora crassa
Source: Biotechnol Biofuels. 2015 Apr 14;8:66. doi: 10.1186/s13068-015-0248-5 (PMC4399147; doi:10.1186/s13068-015-0248-5)
Supplement: Additional file 6: — Supporting information. Additional methods referred to in the main text. [file 13068_2015_248_MOESM6_ESM.doc]

**Additional methods referred to in the main text.**

**Genome-wide analysis of the endoplasmic reticulum stress response during lignocellulase production in *Neurospora crassa***

Feiyu Fan, Guoli Ma, Jingen Li, Qian Liu, J. Philipp Benz, Chaoguang Tian and Yanhe Ma

**1 - Generation of an *N.crassa* *hac-1* deletion strain**

The *hac-1* gene (NCU01856) was deleted following the standard methods provided by the *Neurospora* Functional Genomics Project (<http://www.fgsc.net/ neurosporaprotocols/KO Protocols.pdf>). In order to facilitate genetic assays using the *his-3* locus in future, FGSC9720 instead of FGSC2489 was used as parental strain for *hac-1* deletion in this study. The *hac-1* open reading frame including promoter region (~1.5kb) was replaced by the *hph* (hygromycin B phosphotransferase) marker gene. Successful transformation was confirmed by PCR and Southern blot. For PCR analysis, two PCRs were performed to test whether homologous recombination had occurred as expected (Additional file 8: Figure S3B, D).For Southern blot, genomic DNA was digested with *BglII*, separated by electrophoresis, and transferred to a nylon membrane. The 3’ flanking sequence (left-arm) was subsequently used as a probe (additional file 8: Figure S3B, C). Labeling and visualization were performed using the CDP-Star AlkPhos Direct Labelling Kit following the manufacturer's instructions (GE Healthcare Ltd.).

**2 - Analysis of *hac-1* non-canonical splicing by semi-quantitative RT-PCR**

To amplify *hac-1* splice versions, one microgram of total RNA was reverse-transcribed with iScript^TM^ cDNA synthesis kit (Bio-Rad) and used as PCR templates. The primers used to distinguish *hac-1* splice versions are listed below. PCR products were separated on 2 % agarose gel. The endogenous control *actin* (NCU04173) was used as a loading control. The product sizes representing the *hac-1* spliced and unspliced forms were 201 bp and 224 bp, respectively.

**3 - RNA Sequencing and data analysis**

RNA-seq libraries were generated commercially, and filtered clean reads were aligned to the latest *N.crassa* OR74A genome (version 12) (http://www. broadinstitute.org/annotation/genome/neurospora/MultiHome.html) with splicing aware aligner TopHat2 (version 2.0.12). The annotation file (Broad version 7) was used to improve overall mapping sensitivity and accuracy. Detailed parameters were as follows: no multi-hits (-g 1), minimum anchor length 10 (-a 10), and minimum and maximum intron length of 21 and 4000 bp, respectively. The aligned reads stored in the SAM format file and the raw counts for reads mapping to unique exons were then tallied with HTSeq-count scripts (0.6.0) in intersection-nonempty resolution mode [1] (for paired-end libraries, reads pair counted). After mapping, abundance for each transcript was calculated using the Reads Per Kilobase per Million (RPKM) measure.

Genes with altered expression was determined using R package NOISeq (version 2.6.0) [2, 3]. The extent of gene differential expression was denoted by the”probability of differential expression (q value)” feature which is expressed as a value between 0 and 1. We manually set q value ≥0.95 for single-end libraries and q value ≥0.90 for paired-end libraries as a threshold to select significant expression changes genes, which approximately corresponds to a |log2 ratio|≥1. In order to avoid false positives caused by low-abundance transcripts, genes with expression levels of less than 20 RPKM (single-end) and 15 RPKM (paired-end) [4] were removed.

To reveal potential functional roles of the genes with expression changes, MIPS FunCat online tools (<http://mips.helmholtz-muenchen.de/funcatDB/>) were used to annotate them , and enriched functional categories (P value < 0.01 ) were highlighted. To prove or verify FunCat results these genes were furthermore manually annotated by referring to the functional description of matched orthologues in other model organisms. Homologs of *N.crassa* proteins were identified by using localized Blastp (version Blast + 2.2.28, with e-value < 10^-5^ as cut-off) for each of the following five most intensively studied model organisms: *Saccharomyces cerevisiae* (strain S288C, [http://www.yeastgenome.org](http://www.yeastgenome.org/)); *Aspergillus fumigatus* (Af293, http://www.aspgd. org/,); *Arabidopsis thaliana* ([TAIR10](ftp://ftp.arabidopsis.org/home/tair/Proteins/TAIR10_functional_descriptions), <http://www.arabidopsis.org/>); *Drosophila melanogaster* (Release5.53, <http://flybase.org/>) and *Homo sapiens* (GRCh37.73, <http://asia.ensembl.org/Homo_sapiens/Info/Index>). Domain enhanced lookup time accelerated BLAST (DELTA-BLAST) method was applied due to its advantage in detection of remote protein homologs [5]. Functional categories of differentially expressed genes were divided manually by considering Pfam annotation of *N. crassa*(<http://www.broadinstitute.org/annotation/genome/neurospora/MultiHome.html>) and the function description of matched orthologues.

To identify which ESRTs are regulated by IRE-1, HAC-1, RES-1, RES-2 or RRG-2, we mainly focused on acute ER stress considering that cellulase gene expression only displayed an obvious cross-talk with ER stress under acute conditions. Several criteria were used to profile specific regulons: Firstly, candidate genes must be up-regulated upon ER stress in WT; Secondly, up-regulation upon ER stress in the corresponding transcription factor mutant is absent. Thirdly, candidate genes should belong to the pre-defined ER stress response targets (ESRTs). Finally, to minimize false positive targets, the D value (parameter of NOISeq) fold change was used as additional threshold (|D value ratio|≥1) (See Additional file 5: Table S5 and Additional file 7: Table S7).

**[4] Primers used in cloning and qPCR.**

| **Primer name** | **Sequence(5'-3')** | **Application** |
| --- | --- | --- |
| ***grp-78*-QF** | *GATAAGACTGGTGAGGAG* | qPCR |
| ***grp-78*-QR** | *GGAAAGAAGGGAGACATC* | qPCR |
| ***pdi-1*-QF** | *GTCATTGTTGCCTACCTC* | qPCR |
| ***pdi-1*-QR** | *CCTTGTAGAGAACGATGG* | qPCR |
| ***actin*-QF** | *CGTGGTATCCTTACTCTC* | qPCR |
| ***actin*-QR** | *GTCATCTTCTCACGGTTG* | qPCR |
| ***clr-1*-QF** | *CATGATGACCTCCAACAG* | qPCR |
| ***clr-1*-QR** | *CACTCAGTTCCCTTTGGTC* | qPCR |
| ***clr-2*-QF** | *CTCATCGGTGGTTACTATC* | qPCR |
| ***clr-2*-QR** | *CATATCACTTCCTCCTTGG* | qPCR |
| ***xlr-1*_QF** | *GCTCTCACTATGCTCTTC* | qPCR |
| ***xlr-1*_QR** | *GCTCTGCTTATTCGTCTAC* | qPCR |
| ***cbh-1*-QF** | *CTTCTTCCACCTCTACTG* | qPCR |
| ***cbh-1*-QR** | *CACCAATACCAGCGTTAG* | qPCR |
| ***cbh-2*-QF** | *CAACCATGCCATTCCTTC* | qPCR |
| ***cbh-2*-QR** | *CGTAGACGACAAAGTGAG* | qPCR |
| ***lhs-1*-QF** | *TGCGTCCACCGAATCATC* | qPCR |
| ***lhs-1*-QR** | *GGTGAGGGTGAAATCAAC* | qPCR |
| ***ero-1-QF*** | *GTTTCTCTCCTGCGTAATC* | qPCR |
| ***ero-1-QR*** | *AGGAGGATTTCTGGAAGC* | qPCR |
| ***fpr-2-QF*** | *CTGATTTTCGAGACCGAG* | qPCR |
| ***fpr-2-QR*** | *GCAATCTTTCCTCCAGCC* | qPCR |
| ***hac-1-QF*** | *ATCAACCAGACTCTCCTC* | qPCR |
| ***hac-1-QR*** | *GAGGAGTCGTGCTTTGAC* | qPCR |
| ***ire-1-QF*** | *GAGGACTTCCACGAGATG* | qPCR |
| ***ire-1-QR*** | *CACCATGTTGGTCCTCTTC* | qPCR |
| ***cpc-1-QF*** | *CTGGACCTGCTGGATTTC* | qPCR |
| ***cpc-1-QR*** | *CTAGGAGCAGACAGATAC* | qPCR |
| ***hac-1*_intron_F** | *CAAGACTGAAGAGACCGTC* | Validation of *hac-1* non-canonical splicing by RT-PCR |
| ***hac-1*_intron_R** | *ACTTGGAGCGTGGCACTTC* | Validation of hac-1 non-canonical splicing by RT-PCR |
| ***hph*-F** | *GTCGGAGACAGAAGATGATATTGAAGGAGC* | Construction of *hac-1* KO cassette |
| ***hph*-R** | *GTTGGAGATTTCAGTAACGTTAAGTGGAT* | Construction of *hac-1* KO cassette |
| ***hac-1*-KO5F** | *GTAACGCCAGGGTTTTCCCAGTCACGACGGTCGGTAGAAGGAAGGTTATC* | Construction of *hac-1* KO cassette |
| ***hac-1*-KO5R** | *ATCCACTTAACGTTACTGAAATCTCCAACCCTCGGACCACTCAACATCA* | Construction of *hac-1* KO cassette |
| ***hac-1*-KO3F** | *CTCCTTCAATATCATCTTCTGTCTCCGACCGTGTTAGCGAGTGGTTATC* | Construction of *hac-1* KO cassette |
| ***hac-1*-KO3R** | *GCGGATAACAATTTCACACAGGAAACAGCTCCAGGAGAAGACAGACA* | Construction of *hac-1* KO cassette |
| ***ire-1*-orf-F** | *TGACGGAGATCTGTATGC* | Validation of *ire-1* KO mutant by PCR(Primer Set 1) |
| ***ire-1*-orf-R** | *GATGATCAAGTCGGTGTC* | Validation of *ire-1* KO mutant by PCR(Primer Set 1) |
| ***ire-1*-5out-F** | *AATGGTCATGCAGGCATC* | Validation of *ire-1* KO mutant by PCR (Primer Set 2) |
| ***hph*-check-5R** | *ACCTGCCTGAAACCGAAC* | Validation of *ire-1* KO mutant by PCR (Primer Set 2) |
| ***hac-1*-orf-F** | *ATCGCTCGACGTCATGTCAC* | Validation of *hac-1* KO mutant by PCR (Primer Set 1) |
| ***hac-1*-orf-R** | *GACGGTCTCTTCAGTCTTG* | Validation of *hac-1* KO mutant by PCR (Primer Set 1) |
| ***hac-1*-5out-F** | *TGACGGACACTCAACAGCAG* | Validation of *hac-1* KO mutant by PCR (Primer Set 2) |
| ***hph*-check-5R** | *ACCTGCCTGAAACCGAAC* | Validation of *hac-1* KO mutant by PCR (Primer Set 2) |

**References**

1. Anders S, Huber W: **Differential expression analysis for sequence count data.** *Genome Biol* 2010, **11**:R106.
2. Nookaew I, Papini M, Pornputtapong N, Scalcinati G, Fagerberg L, Uhlén M, Nielsen J: **A comprehensive comparison of RNA-Seq-based transcriptome analysis from reads to differential gene expression and cross-comparison with microarrays : a case study in *Saccharomyces cerevisiae*.** *Nucleic Acids Res* 2012, **40**: 10084-10097.
3. Soneson C, Delorenzi M: **A comparison of methods for differential expression analysis of RNA-seq data.** *BMC Bioinformatics* 2013, **14**:91.
4. McIntyre LM, Lopiano KK, Morse AM, Amin V, Oberg AL, Young LJ, Nuzhdin SV: **RNA-seq : technical variability and sampling**. *BMC Genomics* 2011, **12**:293.
5. Boratyn GM, Schäffer AA, Agarwala R, Altschul SF, Lipman DJ, Madden TL: **Domain enhanced lookup time accelerated BLAST**. *Biol Direct* 2012, **7**:12.
